# Supplementary material for: A high-quality assembled genome and its comparative analysis decode the adaptive molecular mechanism of the number one Chinese cotton variety CRI-12
Source: Gigascience. 2022 Apr 1;11:giac019. doi: 10.1093/gigascience/giac019 (PMC8975723; doi:10.1093/gigascience/giac019)
Supplement: giac019_GIGA-D-21-00277_Revision_2 [file giac019_giga-d-21-00277_revision_2.pdf]

GigaScience

A high-quality assembled genome and its comparative analysis decode the adaptive molecular mechanism of the number one Chinese cotton variety CRI-12

--Manuscript Draft--

**Manuscript Number:**	GIGA-D-21-00277R2
**Full Title:**	A high-quality assembled genome and its comparative analysis decode the adaptive molecular mechanism of the number one Chinese cotton variety CRI-12
**Article Type:**	Research
**Funding Information:**	National Natural Science Foundation of China (32001460) — Prof. Xuke Lu
**Abstract:**	Background: Gossypium hirsutum L. is the most widely cultivated cotton species, and a high-quality reference genome would be a huge boost for researching the molecular mechanism of agronomic traits in cotton.<br>Findings: Here, PacBio and Hi-C sequencing technologies were used to assemble a new upland cotton genome of the number one Chinese cotton variety CRI-12. We generated a high-quality assembled CRI-12 genome of 2.31 Gb with a contig N50 of 19.65 Mb, which was superior to previously reported genomes. Comparisons between CRI-12 and other reported genomes revealed 7,966 structural variations (SVs) and 7,378 presence/absence variations (PAVs). The distribution of the haplotypes among A-genome (G. arboreum), D-genome (G. raimondii), and AD-genome (G. hirsutum and G. barbadense) suggested that many haplotypes were lost and recombined in the process of polyploidization. More than half of the haplotypes that correlated with different tolerances were located on chromosome D13, suggesting that this chromosome may be important for wide adaptation. Finally, it was demonstrated that DNA methylation may provide adaptive advantages in environmental adaptation through whole-genome bisulfite sequencing (WGBS) analysis.<br>Conclusions: This research provides a new reference genome for molecular biology research on Gossypium hirsutum L. and helping decode the broad environmental adaptation mechanisms in the number one Chinese cotton variety CRI-12.
**Corresponding Author:**	Wuwei Ye<br>Institution of Cotton Research of Chiniese Academy of Agricultural Sciences<br>Anyang, Henan CHINA
**Corresponding Author Secondary Information:**	
**Corresponding Author's Institution:**	Institution of Cotton Research of Chiniese Academy of Agricultural Sciences
**Corresponding Author's Secondary Institution:**	
**First Author:**	Xuke Lu
**First Author Secondary Information:**	
**Order of Authors:**	Xuke Lu
	Xiugui Chen
	Delong Wang
	Zujun Yin
	Junjuan Wang
	Xiaoqiong Fu
	Shuai Wang
	Lixue Guo
	Lanjie Zhao

Powered by Editorial Manager® and ProduXion Manager® from Aries Systems Corporation

	Ruifeng Cui
	Maohua Dai
	Cun Rui
	Yapeng Fan
	Yuexin Zhang
	Liangqing Sun
	Waqar Afzal Malik
	Mingge Han
	Chao Chen
	Wuwei Ye
**Order of Authors Secondary Information:**	
**Response to Reviewers:**	Dear editor and two reviewers,<br>Thanks very much for your patience for processing our manuscript entitled "A high-quality assembled genome and its comparative analysis decode the adaptive molecular mechanism of Chinese No.1 cotton variety CRI-12" (GIGA-D-21-00277). We are also very grateful for the two reviewers' hard work on reviewing our manuscript and put forward these valuable suggestions. Now we have revised the manuscript as the comments one by one. The details could be found be below.<br>Reviewer 1:<br>Q1: The authors have nicely addressed all the raised concerns. But, the cited reference for DNA extraction, doesn't seem to be a correct one. Please check, and the relevant DNA extraction methods such as Sahu et al, 2012 (https://pubmed.ncbi.nlm.nih.gov/27335662/) or any other suitable method or kits.<br>Answer: Thanks for the suggestion. We have replaced the reference for DNA extraction with the recommended citation in the manuscript.<br><br>Many thanks again for your patience and consideration.
**Additional Information:**	
**Question**	**Response**
Are you submitting this manuscript to a special series or article collection?	No
**Experimental design and statistics**<br><br>Full details of the experimental design and statistical methods used should be given in the Methods section, as detailed in our Minimum Standards Reporting Checklist. Information essential to interpreting the data presented should be made available in the figure legends.<br><br>Have you included all the information requested in your manuscript?	Yes
**Resources**	Yes

Powered by Editorial Manager® and ProduXion Manager® from Aries Systems Corporation

A description of all resources used, including antibodies, cell lines, animals and software tools, with enough information to allow them to be uniquely identified, should be included in the Methods section. Authors are strongly encouraged to cite Research Resource Identifiers (RRIDs) for antibodies, model organisms and tools, where possible.<br><br>Have you included the information requested as detailed in our Minimum Standards Reporting Checklist?	
**Availability of data and materials**<br><br>All datasets and code on which the conclusions of the paper rely must be either included in your submission or deposited in publicly available repositories (where available and ethically appropriate), referencing such data using a unique identifier in the references and in the “Availability of Data and Materials” section of your manuscript.<br><br>Have you have met the above requirement as detailed in our Minimum Standards Reporting Checklist?	Yes

Manuscript

Click here to access/download;Manuscript;Manuscript-R3_SCE(1).docx

A high-quality assembled genome and its comparative analysis decode the adaptive molecular mechanism of the number one Chinese cotton variety CRI-12

Xuke Lu †, Xiugui Chen†, Delong Wang†, Zujun Yin, Junjuan Wang, Xiaoqiong Fu, Shuai Wang, Lixue Guo, Lanjie Zhao, Ruifeng Cui, Maohua Dai, Cun Rui, Yapeng Fan, Yuexin Zhang, Liangqing Sun, Waqar Afzal Malik, Mingge Han, Chao Chen, Wuwei Ye*

State Key Laboratory of Cotton Biology / Institute of Cotton Research of Chinese Academy of Agricultural Sciences / Research Base, Zhengzhou University / Key Laboratory for Cotton Genetic Improvement, MOA, Anyang, Henan, 455000, China

† These authors contributed equally.

*Corresponding author. The correspondence should be addressed to Wuwei Ye (yew158@163.com), https://orcid.org/0000-0002-0579-4909

ORCID iDs:

Wuwei Ye [0000-0002-0579-4909]; Xuke Lu [0000-0003-4757-1057]; Xiugui Chen [0000-0001-5196-6540]; Delong Wang [0000-0002-4382-0389]; Zujun Yin [0000-0003-4054-681X]; Junjuan Wang [0000-0002-5318-0100]; Xiaoqiong Fu [do not have a ORCID number]; Shuai Wang [0000-0002-7772-6673]; Lixue Guo [0000-0002-8258-7217]; Lanjie Zhao [0000-0003-0991-9024]; Ruifeng Cui [0000-0002-4757-5026]; Maohua Dai [0000-0001-5216-4590]; Cun Rui [0000-0002-6548-1955]; Yapeng Fan [0000-0003-1844-821X]; Yuexin Zhang [0000-0002-0407-4547]; Liangqing Sun [0000-0002-3575-2152]; Waqar Afzal Malik [0000-0001-9562-1624]; Mingge Han [0000-0001-6389-8557]; Chao Chen [0000-0001-7317-4667]

Abstract

Background: *Gossypium hirsutum* L. is the most widely cultivated cotton species, and a high-quality reference genome would be a huge boost for researching the molecular mechanism of agronomic traits in cotton.

Findings: Here, PacBio and Hi-C sequencing technologies were used to assemble a new upland cotton genome of the number one Chinese cotton variety CRI-12. We generated a high-quality assembled CRI-12 genome of 2.31 Gb with a contig N50 of 19.65 Mb, which was superior to previously reported genomes. Comparisons between CRI-12 and other reported genomes revealed 7,966 structural variations (SVs) and 7,378 presence/absence variations (PAVs). The distribution of the haplotypes among A-genome (*G. arboreum*), D-genome (*G. raimondii*), and AD-genome (*G. hirsutum* and *G. barbadense*) suggested that many haplotypes were lost and recombined in the process of polyploidization. More than half of the haplotypes that correlated with different tolerances were located on chromosome D13, suggesting that this chromosome may be important for wide adaptation. Finally, it was demonstrated that DNA methylation may provide adaptive

advantages in environmental adaptation through whole-genome bisulfite sequencing (WGBS) analysis.

Conclusions: This research provides a new reference genome for molecular biology research on *Gossypium hirsutum* L. and helping decode the broad environmental adaptation mechanisms in the number one Chinese cotton variety CRI-12.

Key words: CRI-12, genome assembly, annotation, haplotypes, DNA methylation

Background

Each agricultural crop has their own unique domestication and diversification histories through which their genetic composition was artificially altered, leading to a series of new phenotypic and physiological differences compared with wild types [1]. Upland cotton (*Gossypium hirsutum*, NCBI:txid3635) is not only the most important nature-fiber-producing cotton species worldwide but also an ideal research system for studying polyploidization [2, 3] due to its stronger tolerances to biotic and abiotic environments and high-yield characteristics. *Gossypium hirsutum* L., accounts for over 90% of annual fiber production, and originated from the allopolyplodization event of A-genome-like ancestral species, resembling *Gossypium herbaceum* or *Gossypium arboretum*, and D-genome-like species, resembling *Gossypium raimondii*, approximately 1-2 million years ago (MYA) [4, 5]. The intergenomic interaction in allotetraploid cottons has enabled higher yields, better fiber quality, stronger tolerances and better suiting to mechanization, coincident with the expression bias of these trait-related genes, which provides the preference of selection and domestication of these agronomic traits in cotton [6-8].

To date, much of the genome work on upland cotton has focused on the genetic standard TM-1 and its draft genome and improved genome have been released [1, 5, 9, 10]. In addition, another upland cotton cultivar, the ZM24 (Zhongmiansuo 24) genome, was also assembled and compared with the genetic standard TM-1 to investigate the genetic variations correlated with agronomic traits [11]. Altogether, these genome-assembled technologies and genomic resources offer a series of new opportunities for dissecting the mechanistic basis of primarily agronomic and economic traits.

A haplotype is a set of genes that are linked together at the genome level, which could be inherited by subsequent generations. In our previous research [12], haplotype block inheritance and recombination of agronomically important genes were studied in artificial selection. In cotton evolution, polyploidization played an extremely important role, in addition, the haplotype mechanism in polyploidization was also an important factor. In order to investigate the haplotype mechanism in CRI-12 (Zhongmiansuo 12), a high-quality assembly of the genome is necessary.

CRI-12 (Zhongmiansuo 12) is a well-known cotton cultivar in China for broad adaptation of high-yield, high-quality, multi-resistances to different biotic and abiotic stresses, winning the First Prize of National Invention Award in 1990. It has been the number one cotton cultivar in cotton breeding fields for decades since the commercialization of the CRI-12 variety. In addition, hundreds of new cotton varieties were bred using CRI-12 as one of the two parents, leaving a significant influence on the history of cotton breeding in China and the rest of the world. The planting area of CRI-12 occupied more than 70% of the cotton planting area in China, and it was the number one cotton variety in China in the 1990s. To investigate agronomically important genes in the CRI-12 genome, whole-genome-wide identification and filtration of haplotype blocks correlated with different resistances based on a series of linked genes was reported [12], but this

has been insufficient to answer for many research questions. Therefore, *de novo* assembly of the CRI-12 genome and genome-wide comparative studies are needed to further unravel the genomic components responsible for contrasting traits, providing insights into structural variation and crop improvement. This would also provide a high-quality reference genome for cotton molecular research, especially in China and Asia.

Results

Genomes sequencing and high-quality assembly of CRI-12

The PacBio platform and Hi-C sequencing technology were selected to perform chromosome-scale assembly for *Gossypium hirsutum* L. CRI-12 (**Fig. 1a and Supplementary Fig. S1**), which was bred in fusarium wilt and verticillium wilt nursery for many years through the crossing of Uganda4 and Xingtai687 cultivars. CRI-12 has become the most widely planted cotton variety in China from 1989 to the present due to its excellent performance (**Supplementary Table S1**) in three major cotton-production regions, including the Yangtze, the Yellow River and Inland regions. In total, we produced ~ 264 Gb of high-quality data, and the sequencing depth reached 110.94× (the estimated genome size was 2379.62M). In addition, a second-generation small fragment library was constructed and sequenced with an insert size of 350 bp using the Illumina platform to assist genome assembly, and 53 Gb of data was generated with an average read length of 150 bp (~ 22.27 coverage) (**Supplementary Table S2**). Approximately 264 Gb PacBio reads (~110.94 coverage) were obtained to assemble the CRI-12 genome. After correction using the Illumina short reads, we generated a CRI-12 genome of 2.31 Gb with a contig N50 of 19.65 Mb (**Table 1 and Supplementary Table S3**). Total scaffold length was 2199.32 Mb and the length of scaffold N50 reached 91.74 Mb.

The results indicated that the GC content was 34.34% and the ratio of N was 0.00%, suggesting that the ratio of four bases was correct (**Supplementary Table S4 and Supplementary Fig. S2**). In addition, Hi-C libraries which have been widely used to aid the assembly of contigs on chromosomes [13-15] were used. The results showed that approximately 98.55% of 2.31 Gb of data were successfully oriented, and from this data were organized into 26 chromosomes (**Fig. 1b** and **Supplementary Table S5**). In comparison with several recently reported genome assemblies for *Gossypium hirsutum* L., the updated CRI-12 genome showed higher contiguity and quality (19.65 Mb versus 15.51 Mb [1], 4.8 Mb [11] and 2.1 Mb [11], 91.74 Mb versus 48 Mb [1]. BUSCO (version: 5.2.1) assessment was also used to estimate the integrity of the CRI-12 genome, and the results showed that 99.60% of the complete single-copy genes were assembled from 1614 orthologous homologous single-copy genes, indicating that the assembly result was relatively complete (**Supplementary Table S6**). LAI (LTR Assembly Index) was also used to assess the integrity of CRI-12 genome assembly, and the LAI score was 14.39, indicating CRI-12 genome can be used as a reference genome.

Synteny analysis of different cotton species indicated that both A and D subgenome showed high collinear relationships (**Fig. 1c**). Phylogenetic and evolutionary analysis of CRI-12 genome showed both A subgenome and D subgenome derived from *Gossypium hirsutum* L., but the relationship between D subgenome and A subgenome was different in different cotton species (**Supplementary Fig. S3).** Using the orthologous gene pairs of *Gossypium hirsutum* L. CRI-12 and other species, including *Arabidopsis*, Coffee and Durio, identified by gene collinearity and paralogous pairs identified by gene clustering, 4DTv (4-fold degenerate synonymous sites of the

third codons) values were calculated for all of the duplicated pairs (**Fig. 1d**). A relatively close species divergence peak (4DTv ~ 0.15) was observed between CRI-12 and Durio, while larger divergences were found between CRI-12 and *Arabidopsis* (4DTv ~ 0.55) and Coffee (4DTv ~ 0.65).

Annotation analysis of CRI-12 genome

Building upon the high-quality assembly of the CRI-12 genome, detailed annotations were performed (**Fig. 2**). In the CRI-12 genome, annotation results showed that a total of 72,293 genes were obtained with multiple prediction tools (**Table 2 and Supplementary Fig. S4**). Compared with previous reports, the number of predicted genes in the CRI-12 genome was slightly greater than that in reported Island cotton Hai7124, and less than that in upland cotton TM-1 and ZM24, which may be correlated with the higher integrity and continuity of the CRI-12 genome. We also compared the different elements in proximal species (**Supplementary Fig. S5**).

Among all predicted genes, it was found that the average gene length, CDS length, number of exons per gene, exon length and intron length were 2834 bp, 1134 bp, 4.98, 227 bp and 427 bp, respectively. Approximately 99.30% of CRI-12 genes were functionally annotated and shared homology with already known genes in the Swiss-Prot, Nr, KEGG, InterPro, GO and Pfam databases (**Supplementary Table S7 and Fig. 3a)**. Repeat sequences are widely distributed throughout most plant genomes and play a vital role in genome divergence [16]. Overall, approximately 63.55% of the assembly sequences were annotated as repeat sequences with the RepeatMasker program based on the repeat database predicted by De *novo* and the homologous repeat database predicted by RepBase (**Supplementary Table S8**), which was a slightly lower than that in TM-1 and ZM24. Among all repeat sequences, 62.57% were TEs, including DNA transposons, LINEs, SINEs, LTRs and some other unknown TEs (**Supplementary Table S9**). LTR transposons were the largest category with a ratio of 93.06% among all TE transposons while SINE transposons accounted for only 1.60%. In addition, based on the Repbase protein database, the degree of ramification of TEs derived from the genome assembly of CRI-12 and the sequences in the Repbase database was investigated **(Supplementary Fig. S6**), and the results indicated that the degree of ramification of TEs between them conforms to a normal distribution as a whole.

Furthermore, we examined the number of non-coding RNAs including miRNAs, tRNAs, rRNAs, and snRNAs (snRNA, CD-box, HACA-box, splicing), in the CRI-12 genome and finally identified 523, 2 214, 2 749, 8 160 miRNAs, tRNAs, rRNAs and snRNAs, respectively (**Supplementary Table S10**). The total length of miRNAs was 67,890 bp, and the average length was 129 bp, while the total length of tRNAs was 166,317 bp, and the average length was 75 bp. In addition, the total length of snRNAs was the longest among of all non-coding RNAs with 882 686 bp, while the average longest non-coding RNAs were rRNAs, and the average length was 254 bp, which may be correlated with the specific functions of rRNAs in the process of protein translation and modification.

Expanded and contracted gene families related to stress tolerance in CRI-12

To reveal the genetic basis underpinning *Gossypium hirsutum* L. CRI-12, we investigated the number and evolution of gene families that were unique or shared among different cotton species. Gene families are frequently derived from the same ancestor, undergoing a series of gene duplications and species differences of two or more copies and sharing obvious similarities in

structure, function and protein products. Identification and annotation analysis of gene family clustering is an important aspect of evolutionary analysis, which is also associated with biological characteristics. In our research, 22,854 gene families were shared by CRI-12, *Gossypium hirsutum* TM-1, *Gossypium barbadense*, *Gossypium mustelinum* and *Gossypium darwinii*, while 555 gene families were retained only by CRI-12 (**Fig. 3b)**. Based on the gene family clustering analysis, in order to investigate the expansion and contraction of gene members in CRI-12 and other cotton species, six gene families, included MYB, WRKY, DREB, bZIP, NAC and AP2, were finally selected. The results showed three gene families were expanded and 40 gene families were contracted among 1,188 gene families shared in MRCA (most recent common ancestor) analysis (**Fig. 3c**). Among the three expanded gene families, 36 genes were discovered, mainly located on chromosomes A08, D08, A11 and D11. The discovery of abundant stress-related gene families and genes demonstrates the utility of the newly assembled CRI-12 genome as a reference genome for research on stress-related molecular biology.

Positive selection refers to a single copy gene family, in which a gene is affected by environmental or human factors in the process of evolution, and non-synonymous mutation occurs at the amino acid level to adapt to environmental changes. The probability of positive selection is detected by calculating Ka/Ks using the maximum likelihood ratio. In this study, CRI-12 was used as the foreground branch, and upland cotton, island cotton, wool cotton, yellow brown cotton and Darwin's cotton were used as the background branches. Multiple sequence alignments of protein sequences from single copy gene families were performed using MUSCLE software. For each gene family, the branch-site model of the Codeml tool in PAML (phylogenetic analysis by maximum likelihood, a package of programs for phylogenetic analyses of DNA and protein sequences using maximum likelihood) was used to detect whether the gene family was positively selected in the CRI-12 branch. In PAML, instead of simply searching for genes with the Ka/Ks ratio >1, positive selection is determined by likelihood ratio tests of the two hypotheses. Finally, by likelihood ratio detection, 384 candidate genes were identified in CRI-12 (**Fig. 3d** and **Supplementary Table S11)**. Based on the selected genes, results with a P value < 0.05 were filtered out according to Fisher's exact test, and 63 and 27 significant pathways were obtained by Gene Ontology (GO) and KEGG enrichment analysis, respectively (**Supplementary Table S12 and Supplementary Table S13)**. One of the enriched GO terms "intracellular" (GO: 0005622, P $\ll$ 0.01) contains many stress-related genes, including many MYB transcription factors [17], cytochrome P450 genes [18], and E3 ubiquitin-protein [19], which were reported to be closely correlated with multiple tolerances in cotton. The results showed that many genes correlated with the cell membrane were reasonably important for environmental adaptation.

Major structural changes compared with other cultivated genomes

High-quality reference genomes provide a basis for accurate genome-wide structural variations, which are closely correlated with multiple agronomic traits between different species. In our research, a total of 7,966 structural variations (SVs) were identified with an average length of 48,791 bp compared with other cotton species, including *Gossypium hirsutum* TM-1, *Gossypium barbadense*, *Gossypium mustelinum* and *Gossypium darwinii* (**Supplementary Table S14**). Among all SVs, 46.16% (3 677) were deletion variations (DELs) and 40.57% (3 232) were insertion variations (INS), while only a small proportion of 8.29% and 4.98% were copy number variants (CNV) and inversion variations (INV), respectively, which suggested that deletion

variations and insertion variations were two main drivers in the differentiation process of cotton species. The largest structural variation was located on chromosome D11. In addition, 7,379 PAVs were obtained with the lengths ranging from 51 bp to 2,452,232 bp and the average length was 24,585 bp (**Supplementary Table S15**). The largest PAV was located on the D02 chromosome. The results also showed that the percentage of variations of both SVs and PAVs on the Dt subgenome was lower than that on the At subgenome. In particular, there were lesser PAV variations (13.80%) in the Dt subgenome than that in the At subgenome, indicating that variations in the A subgenome was the main reason for the difference in agronomic characteristics.

We also investigated the SV and PAV variations on each chromosome, and the results showed that chromosome D01 covered 529 SVs (7.17%, mainly containing copy number variants, deletion variations, insertion variations and inversion variations) and 363 PAVs (4.56%), which was the most compared with PAVs on other chromosomes. GO enrichment analysis of PAV-related genes (**Fig. 4a**) showed that molecular transducer activity (GO: 0060089, $p<0.01$), signaling receptor activity (GO: 0038023, $p<0.05$), and the G-protein-coupled receptor signaling pathway (GO: 0007186, $p<0.05$) were three main terms while SV-related genes (**Fig. 4b**) were mainly enriched in organelle (GO: 0043226, $p<0.01$), intracellular non-membrane-bounded organelle (GO: 0043232, $p<0.001$), and non-membrane-bounded organelle (GO: 0043228, $p<0.0001$), which were all belonged to cellular components. Pathway enrichment analysis of SV- and PAV- related genes showed that most variation-related genes were correlated with organelles, signaling receptor and molecular transducers, indicating that the evolution and differences of organelles, signal reception and transduction related genes may be important factors leading to the great differences of agronomic traits among different cotton varieties (**Fig. 4c, d**). In contrast, several chromosomes contained less variation, e.g., D03 (138 SVs and 124 PAVs), D04 (169 SVs and 115 PAVs) and D13 (189 SVs and 129 PAVs), which indicated that two chromosomes may be conserved for containing many fundamental growth related genes in the long-term evolution process of cotton.

Based on our previous research, 420 genes were obtained by selective sweep analysis in CRI-12, including 2, 2 and 20 haplotype blocks correlated with *V. wilt*, salt- and drought-tolerance, respectively [12]. Among these haplotype blocks, more than half (13/24) were located on chromosome D13. Interestingly, these 12/13 haplotype blocks were correlated with drought-tolerance (**Supplementary Table S16**), indicating that D13 chromosome played a crucial role in the process of drought resistance adaptation of cotton varieties. In addition, another haplotype block (M2: ATCTCGCATGTAGAGTTCAT CCGGTAGAAACCGTTTTACAT) was also found to be correlated with *Verticillium wilt*, suggesting that chromosome D13 may be important for the formation process of multiple tolerances.

Strong haplotypes were discovered in the polyploidization and evolution of diploid cottons

A haplotype means a group of genes that have a close linkage relationship in an organism and these haplotypes could be inherited from parents to their descendants. In our previous research, haplotype polymorphisms in CRI-12 and its descendants and different reported genomes (*G. arboreum*, *G. raimondii*, *G. hirsutum*, and *G. barbadense*) were investigated [12]. All allotetraploid cotton species came from a single polyploidization event between the A-genome and D-genome approximately 1-2 million years ago [11, 20]. In the polyploidization process, two diploid genomes were hybridized into a tetraploid genome, along with the fusion and recombination of haplotypes in each diploid cotton. With that in mind we investigated the

haplotype polymorphisms in the A-genome (*G. arboreum*), D-genome (*G. raimondii*), TM-1 (*G. hirsutum*), and Hai7124 (*G. barbadense*), and obtained 31 769, 37,177, 51,682, 51,023 haplotypes, respectively (**Supplementary Table S17)**. A total of 56,267 haplotypes were discovered in CRI-12, which was the highest number in the different cotton species. Alongside *G. hirsutum* and *G. barbadense*, in CRI-12 the number of haplotypes was smaller than the sum of the A-genome (*G. arboreum*) and D-genome (*G. raimondii*), indicating that more than 10,000 haplotypes were lost or recombined in the process of polyploidization (**Fig. 5**). Comparisons between *G. hirsutum* CRI-12 and *G. barbadense* Hai7124 showed that the number of haplotypes between *G. hirsutum* CRI-12 and *G. barbadense* Hai7124 was approximately 10% more than that between *G. hirsutum* TM-1 and *G. barbadense* Hai7124, suggesting that the haplotype polymorphism in CRI-12 was more abundant than other tetraploid cotton species, which may be correlated with a great deal of human selection and strong haplotypes in the breeding process in CRI-12.

DNA methylation may provide adaptive advantages in broad environmental adaptation in CRI-12

DNA methylation variations are the most common epigenetic modification and are closely correlated with normal growth and development, organ differentiation, stress responses, and environmental adaptation [21]. To investigate whether DNA methylation was involved in the formative process of important agronomic traits in CRI-12, whole-genome wide bisulfite sequencing (WGBS) of leaves under drought and salt stress was performed. In our previous results, we revealed the haplotype inheritance and recombination of agronomically important genes in artificial selection [12], and combined with the haplotypes identified before, a total of 66 differentially methylated haplotypes were found in the CRI-12 family (**Supplementary Table S18)**. Among these haplotypes, six were derived from its female parent Uganda4 and 19 haplotypes were derived from its male parent Xingtai6871, indicating that the greater contribution of DNA methylation haplotypes was from the male parent Xingtai6871 as it was a domestic variety while Uaganda4 was a foreign variety. Approximately 12.12% (8/66) of DNA methylation haplotypes were enriched on chromosome D13, suggesting that DNA methylation variations on D13 may play a crucial role in the regulatory mechanism of haplotypes in CRI-12. In addition, methylation types in each haplotype were studied, and the results showed that six haplotypes were labeled as CG-up methylation under both drought and salt treatment **(Supplementary Fig. S7**), which indicated that DNA methylation variations in these haplotypes may provide adaptive advantages in responding to different stresses (**Fig. 6)**.

Discussion

In this study, we performed *de novo* assembly of the CRI-12 genome by integrating multiple sets of data from the PacBio platform, 110× genome equivalent sequencing, and Hi-C technology. All these results indicated substantial improvements to the contiguity and accuracy of assembly, with a significant enhancement in the assembly of centromeres. By comparing the two high-quality genome assemblies (CRI-12 and *Gossypium hirsutum* ZM24), 7,966 SVs (accounting for 12.65% of the assembled genome) and 7,378 PAVs (accounting for 17.85% of the assembled genome) were obtained between different species, demonstrating these large variations projected differences in traits and species differentiation. Structural variations are generally considered to be relatively large variations and stable, hence, SV-related genes may be the main cause for the

differences in characteristics. Chromosomes D03, D04 and D13 contained fewer than 200 SVs, significantly lower than other chromosomes, suggesting that these chromosomes are relatively conserved. Cotton polyploidization was a crucial event in cotton history, and tracking the haplotype mechanism is beneficial for understanding the cotton evolution. In addition, strong haplotypes contained in *G. hirsutum* CRI-12 suggested intense human selection and domestication occurred during the breeding process.

CRI-12, a cotton variety well known by every cotton breeder in China, have been repeatedly used for breeding new cotton varieties, and haplotype blocks inheritance and recombination of agronomically important genes is likely one of the main reasons that CRI-12 is such an effective breeding parent. The whole-genome scale methylation map of CRI-12 suggested that DNA methylation variations may be closely correlated with the haplotype block inheritance and recombination. To our knowledge, this is the first genome map of the widely cultivated upland cotton CRI-12, which could provide more understanding of crop domestication, evolution and diversification and the discovery of novel domestication-related genes conferring agronomically beneficial traits in future breeding programs. In addition, it demonstrates the suitability of selecting the CRI-12 genome as a new reference genome for its many suitable indicators.

Conclusion

In this study multiple sequencing techniques and analytical methods were used to assemble a new upland cotton genome of the most popular Chinese cotton variety CRI-12. The newly assembled CRI-12 genome was approximately 2.30 Gb in length and several benchmarks reveal its improved quality to other reported genomes. This research providing a better quality alternative for cotton researchers when selecting a reference genome.

Materials and methods

Plant materials

G. hirsutum L. acc. CRI-12 was selected for genome assembly because of its excellent performance and wide influence on cotton breeding and genetic research in China and many other cotton-growing countries across the globe. CRI-12 seeds were planted in the greenhouse for 20 days at the Institute of Cotton Research of Chinese Academy of Agricultural Science and young leaves from a single plant were harvested and snap frozen with liquid nitrogen for the extraction of genomic DNA [22]. In addition, root, stem and flower tissues were used for transcriptome sequencing for genome annotation work using three replicates.

PacBio sequencing

Genomic DNA of CRI-12 was extracted using an improved CTAB method utilizing phenol/chloroform/isoamyl alcohol (PCI) solution (25:24:1), DNase (RNase- and Protease-Free - Molecular Biology grade), pH 7.8-8.2, Chloroform/isoamyl alcohol, 24:1 (Molecular Biology grade), Elution Buffer (10 mM Tris-HCl, pH 8.5), NH_4OAc, concentrated solution, Glycogen, 20 mg/ml, 5% (w/v) PVP40 and β- mercaptoethanol. RNA polymerase (10 mg mL^{-1}) was added to remove the residual RNA. Genomic DNA degradation and purity were checked with 1.0% agarose gels and a Nanodrop 2000 to ensure high-quality DNA for sequencing. The DNA/RNA libraries were sequenced using the PacBio sequencing platform by LC-Bio Technology Co., Ltd (Hang Zhou, China) (PacBio Sequel System, RRID:SCR_017989).

Annotation of repeats

After genome assembly, repeat annotation was performed for the CRI-12 genome. Repetitive sequences include transposable elements (TEs) and tandem repeats. Two approaches were used to discover TEs. RepeatMasker (RepeatMasker, RRID:SCR_012954) (version 3.3.0) [23] found TEs in an integrated repeat library derived from a known repeat library (Repbase 15.02) and the *de novo* repeat library, built by RepeatModeler1 (RepeatModeler, RRID:SCR_015027) (Vision 1.0.5), RepeatScout (RepeatScout, RRID:SCR_014653) [24], Piler (PILER, RRID:SCR_017333) and LTR_FINDER (LTR_Finder, RRID:SCR_015247) [25]. Repeat ProteinMask [23] was performed to detect TEs in the CRI-12 genome by comparing the TE protein database. Tandem repeats were ascertained in the genome using Tandem Repeats Finder (TRF) [26]. The results showed that repetitive sequences comprised 63.55% of the CRI-12 genome.

Hi-C experiment

Leaves were fixed with 1% formaldehyde solution in MS buffer (10 mM potassium phosphate, pH 7.0; 50 mM NaCl; 0.1 M sucrose) at room temperature for 30 min in a vacuum. After fixation, the leaves were incubated at room temperature for 5 min under vacuum in MC buffer with 0.15 M glycine. Approximately 2.0 g of fixed leaves were homogenized with liquid nitrogen, resuspended in nuclei isolation buffer and filtered with a 40-nm cell strainer. The procedures for enriching nuclei from flow-through and subsequent denaturation were performed according to a 3C protocol established for maize.

The chromatin extraction method was similar to that used in the DNase I digestion experiment. The procedures were similar to those described previously. Briefly, chromatin was digested for 16 h with 400 U HindIII restriction enzyme (NEB) at 37 °C. DNA ends were labeled with biotin and incubated at 37 °C for 45 min, and the enzyme was inactivated with 20% SDS solution. DNA ligation was performed by the addition of T4 DNA ligase (NEB) and incubation at 16 °C for 4~6 hours. After ligation, proteinase K was added to reverse cross-linking during incubation at 65 °C for overnight. DNA fragments were purified and dissolved in 86 μL of ultrapure water. Unligated ends were then removed. Purified DNA was fragmented to a size of 300-500 bp, and DNA ends were then repaired. DNA fragments labeled by biotin were finally separated on Dynabeads® M-280 Streptavidin (Life Technologies). Hi-C libraries were controlled for quality and sequenced on an Illumina HiSeq X Ten sequencer (Illumina HiSeq X Ten, RRID:SCR_016385).

Hi-C library preparation and sequencing

Following the standard protocol previously described with certain modifications [27], we constructed Hi-C libraries using the CRI-12 seedlings as inputs (20-day-old, detailed culturing conditions were described in the main text). After being ground in liquid nitrogen, seedling tissues were cross-linked with 4% formaldehyde solution at room temperature in a vacuum for 30 mins. 2.5 M of glycine was added to quench the crosslinking reaction for 5 min and then placed on ice for 15 min. The sample was centrifuged at 2500 rpm at 4 °C for 10 min, and the pellet was washed with 500 μl of PBS and then centrifuged for 5 min at 2500 rpm. The pellet was resuspended in 20 μl lysis buffer (1 M Tris-HCl, pH 8.0, 1 M NaCl, 10% CA-630, and 13 units protease inhibitor), and then the supernatant was centrifuged at 5000 rpm at room temperature for 10 min. The pellet was washed twice in 100 μl of ice cold 1x NEB buffer and then centrifuged for 5 min at 5000 rpm.

The nuclei were resuspended in 100 μl of NEB buffer and solubilized with dilute SDS followed by incubation at 65 °C for 10 min. After quenching the SDS with Triton X-100, a 4-cutter restriction enzyme *Mbo*I (400 units) was applied for overnight digestion at 37 °C on a rocking platform.

The following steps were involved in marking the DNA ends with biotin-14-dCTP and blunt-end ligation of the cross-linked fragments. The proximal chromatin DNA was religated by ligation enzyme. The nuclear complexes were reversely cross-linked by incubation with proteinase K at 65 °C. DNA was purified by phenol-chloroform extraction. Biotin was removed from nonligated fragment ends using T4 DNA polymerase. Ends of fragments sheared by sonication (200-600 base pairs) were repaired by the mixture of T4 DNA polymerase, T4 polynucleotide kinase and Klenow DNA polymerase. Biotin-labeled Hi-C samples were specifically enriched using streptavidin C1 magnetic beads. After adding A-tails to the fragment ends and the following ligation with the Illumina paired-end (PE) sequencing adapters, Hi-C sequencing libraries were amplified by PCR (12-14 cycles) and sequenced on an Illumina HiSeq-2500 platform (PE 125 bp).

Hi-C assisted assembly

The Hi-C technique obtains information about interactions between DNA fragments that are spatially connected, that is, physically distant. Different contigs or scaffolds were divided into different chromosomes according to the rule that the interaction probability within chromosomes was significantly higher than the interaction probability between chromosomes. The contigs or scaffolds on the same chromosome are sequenced and orientated base on the interaction probability decreasing with the increase of the interaction distance on the same chromosome.

(1) Comparison with the draft genome. Effective high-quality sequencing data were compared to the draft genome by BWA (BWA, RRID:SCR_010910) [28] software, and the comparison results were removed by SAMTOOLS (SAMTOOLS, RRID:SCR_002105) [29] to obtain the high quality data. Meanwhile, reads near the enzyme cutting sites were extracted for auxiliary assembly.

(2) Clustering. Firstly the short reads obtained by sequencing were compared to the draft genome. Then the number of interactions between contigs was counted, and contigs were clustered according to the number of interactions and divided into specified groups according to the number of chromosomes of species.

(3) Ranking and orientation. According to the results of clustering, ranking and orientation were conducted according to the strength of the interactions of two contigs and the location of the reads comparison.

Sequence quality checking and filtering

To avoid reads with artificial bias, we removed the following type of reads: (a) reads with ≥ 10% unidentified nucleotides (N); (b) reads with > 10 nt aligned to the adapter, allowing ≤ 10% mismatches; (c) reads with > 50% bases having phred quality < 5; and (d) putative PCR duplicates generated by PCR amplification in the library construction process.

Haplotype analysis (alignment, variant calling, HapCUT analysis)

The high-quality paired-end Hi-C reads were first mapped to the reference genome using Burrows-Wheeler Aligner (BWA) software [28]. Alignment files were converted to BAM files using SAMtools [29], and then the alignment results were improved as follows: (a) filter the

alignment read with mapping quality = 0; (b) sort the BAM files as physical coordinate; (c) remove potential PCR duplications. If multiple read pairs have identical external coordinates, only the pair with the highest mapping quality retained. (d) Local InDel realignment was performed.

The filtered BAM files of CRI-12 leaves were used as input for variant calling using the Genome Analysis Toolkit version 3.1.1 (GATK) [30]. SNPs and InDels were remained if the depth of alternative variants was above 2 and the genotype quality was more than 20.

We used the modified version of the HapCUT [31] algorithm to perform haplotype imputation for each individual. HapCUT constructs a graph with the heterozygous variants as nodes and DNA fragment (s) connecting two nodes as edges. Therefore, only fragments with at least two heterozygous variants are useful for haplotype phasing. HapCUT extracts such "haplotype-informative" BAM files using a sorting method that stores each potential haplotype-informative read in a buffer until its mate is seen. We set 30 Mb as the maximum "insert size" for a paired-end read to be considered as a single fragment for phasing. HapCUT uses a greedy max-cut heuristic to identify the haplotype solution for each connected component in the graph with the lowest score under the MEC scoring function. As Hi-C data result in chromosomal spanning haplotypes with a single large connected component, the higher the number of heterozygous variants in the largest connected component of the graph, the lower this parameter. We used a maximum of 1000 iterations to find the maximum cut for each haplotype block in a given iteration in CRI-12.

Hi-C read mapping and filtering and generation of contact matrices

For the Hi-C experiment, chromatin was crosslinked with formaldehyde, then digested, and religated to capture 3D interactions. In principle, interactions within chromosomes are more frequent than those among chromosomes, and the intrachromosome interaction frequency decays with the increasing distance. Contigs/scaffolds are thus ordered and oriented. The high quality paired-end Hi-C reads were mapped to mm10 and filtered using HiCUP (HiCUP, RRID:SCR_005569) [32]. The first stage in the HiCUP pipeline involves truncating reads at the enzyme digestion ligation site (HindIII in our experiment) that separates two DNA fragments. After truncation, the resulting trimmed forward and reverse reads were sent for alignment by Bowtie2 software (Bowtie 2, RRID:SCR_016368) [33]. These unique high-quality alignments were remained for further analysis. HiCUP removes sequences representing experimental Hi-C artifacts and other uninformative di-tags, since even a small number of invalid di-tags could lead to incorrect conclusions being drawn concerning genomic structure.

The genome was divided into 1 Mb bins, and the read pair numbers in two regions were counted as the observed interactions. The observed matrix was normalized for GC content near the ligated fragment ends, fragment lengths digested by HindIII and the mappability of the fragment ends [34]. Through normalization, we obtained the expected interactions between every two bins. The norm interactions were computed by observed interactions divided by expected interactions. We used the norm interactions for every two bins to produce a norm contact matrix.

mRNA library construction and sequencing

Total RNA was isolated and purified using TRIzol reagent (Invitrogen, Carlsbad, CA, USA) in accordance with the manufacturer's instructions. The amount and purity of RNA for each sample was quantified using a NanoDrop ND-1000 (NanoDrop, Wilmington, DE, USA). RNA integrity

was then assessed using a Bioanalyzer 2100 (Agilent, CA, USA) with RIN number >7.0, and confirmed by electrophoresis using a denaturing agarose gel. Poly (A) RNA was purified from 1μg of total RNA using two rounds of Dynabeads Oligo (dT) 25-61005 (Thermo Fisher, CA, USA) purification. The resulting poly (A) RNA was then fragmented using Magnesium RNA Fragmentation Module (NEB, cat.e6150, USA) at 94 ℃ for 5-7 min. The cleaved RNA fragments were then reverse transcribed to generate the cDNA using SuperScript™ II Reverse Transcriptase (Invitrogen, cat. 1896649, USA), which were next used to synthesize U-labeled second-stranded DNAs with *E. coli* DNA polymerase I (NEB, cat.m0209, USA), RNase H (NEB, cat.m0297, USA) and dUTP Solution (Thermo Fisher, cat.R0133, USA).

A-base was added to the blunt ends of each strand for ligation to the indexed adapters. Each adapter contained a T-base overhang to allow ligation of the adapter to the A-tailed fragmented DNA. Single- or dual- index adapters were then ligated to the fragments, and size selection was performed using AMPureXP beads. After heat-labile UDG enzyme (NEB, cat.m0280, USA) treatment of the U-labeled second-stranded DNAs, the ligated products were amplified via PCR using the following conditions: initial denaturation at 95 ℃ for 3 min; 8 cycles of denaturation at 98 ℃ for 15 sec, annealing at 60 ℃ for 15 sec, and extension at 72 ℃ for 30 sec; and a final extension at 72 ℃ for 5 min. The average insert size for the final cDNA library was 300±50 bp. Finally, we performed 2×150 bp paired-end sequencing (PE150) using an Illumina NovaSeq™ 6000 (LC-Bio Technology CO., Ltd., Hangzhou, China) (Illumina NovaSeq 6000 Sequencing System, RRID:SCR_016387) following the vendor's recommended protocol.

mRNA Sequence and primary analysis

Cutadapt software (version: cutadapt-1.9) was used to remove the reads that contained adaptor contamination (command line: ~ cutadapt - a ADAPT1 -A ADAPT2 -o out1.fastq -p out2.fastq in1.fastq in2.fastq -O 5 -m 100). After the removal of the low quality and undetermined bases, we used HISAT2 software (version: hisat2-2.0.4, RRID:SCR_015530) [35] to map reads to the genome (for example: Homo sapiens Ensembl v96), (command line: ~hisat2 -1 R1.fastq.gz -2 R1.fastq.gz -S sample_mapped.sam). The mapped reads of each sample were assembled using StringTie (version: stringtie-1.3.4d.Linux_x86_64) [36] with default parameters (command line: ~ stringtie -p 4 -G genome.gtf -o output.gtf -l sample input.bam). Then, all transcriptomes from all samples were merged to reconstruct a comprehensive transcriptome using gffcompare software (version: gffcompare-0.9.8. Linux_x86_64). After the final transcriptome was generated, StringTie (StringTie, RRID:SCR_016323) and Ballgown were used to estimate the expression levels of all transcripts and mRNAs by calculating FPKM (FPKM = [total_exon_fragments / mapped_reads (millions) × exon_length (kB)]), (command line: ~ stringtie -e -B -p 4 -G merged.gtf -o samples.gtf samples.bam). The differentially expressed mRNAs were selected with fold change > 2 or fold change < 0.5 and p value < 0.05 using the R package edgeR [37] or DESeq2 and then GO and KEGG enrichment to the differentially expressed mRNAs [38, 39].

Generation of inter-chromosomal contacts matrix

The expected number of inter-chromosomal interactions for each chromosome pair *i,j* was computed by multiplying the fraction of inter-chromosomal reads containing *i* with the fraction of inter-chromosomal reads containing *j* and multiplying by the total number of inter-chromosomal reads. The enrichment was computed by taking the actual number of interactions observed

between *i* and *j* and dividing it by the expected value.

The inter-chromosomal contact possibility was computed by the observed read pairs between chromosome pair *i,j* dividing it by its expected value. The expected number of inter-chromosomal interactions for each chromosome pair *i,j* was calculated by multiplying the proportion of inter-chromosomal reads containing *i* with the proportion of inter-chromosomal reads containing *j* and the total number of inter-chromosomal reads. In addition, based on colinear mapping results, comparative genome analysis between CRI-12 and other published upland genomes was performed to investigate different types of structure variations (SVs). Enrichment analysis of SV-related genes was also performed, including GO and KEGG enrichment.

Promoter associated interactions statistics

The Eukaryotic Promoter Database (EPD) is a collection of databases of experimentally validated promoters for selected model organisms. We downloaded 21,239 mouse TSS sites from the EPD database in the mm9 genome version. We considered the region from 1,000 bp upstream to 100b downstream of the TSS site as the promoter region. These promoter sequences were subsequently mapped to mm10 and retained unique alignment to obtain the promoter region in the new genome version. After alignment, 21,226 promoters were left for further statistical analysis. We counted the interaction numbers in promoter-promoter, promoter-other and other-other.

Annotation of protein coding genes

De novo, homolog-based and RNA-seq based predictions were employed to annotate the protein coding genes in the CRI-12 genome. Five *ab initio* gene prediction programs were used to predict genes, including Augustus [40, 41] (version 3.0.2, RRID:SCR_008417), Genescan [42] (version 1.0), Geneid [43], GlimmerHMM [44] (version 3.0.2, RRID:SCR_002654) and SNAP [45]. Protein sequences of six (five) homologous species (*Arabidopsis thaliana*, *Oryza sativa.*, *etc.*) were downloaded from Ensembl or NCBI. Homologous sequences were aligned against the repeat-masked CRI-12 genome using TBLASTN [46] (E-value ≤ 1E-05, RRID:SCR_011822). Genewise [47] (version 2.2.0, RRID:SCR_015054) was employed to predict gene models based on the alignment sequences. There were two ways to assemble the RNA-seq data into the unique sequences of transcripts. One was mapping the RNA-seq data to the CRI-12 genome using TopHat [48] (version 2.0.8, RRID:SCR_013035) and using cufflinks [49] (version 2.1.1, RRID:SCR_014597) for transcript assembly. The other was applying Trinity [50] to assemble the RNA-seq data, and then PASA [51] software improved the gene structures. A weighted and non-redundant gene set was generated by EVidenceModeler (EVM) (EVidenceModeler, RRID:SCR_014659) [52], which merged all gene models predicted by the above three approaches. Combined with transcript assembly, PASA adjusted the gene models generated by EVM. The final reference gene set contained 72,293 protein coding genes.

Functional annotation

Functional annotation of protein coding genes was obtained according to the best BLAST hit by BLASTP (E-value ≤ 1E-05, RRID:SCR_001010) against the SwissProt, TrEMBL [53] and NCBI non-redundant (NR) protein databases. Motifs and domains were annotated using InterProScan [54] (version 4.7, RRID:SCR_005829) to search against InterPro [54] (v29.0) databases, including Pfam, PRINTS, PROSITE, ProDom and SMART. A Gene Ontology [55] (GO) term for each gene

was obtained from the corresponding InterPro descriptions. Additionally, the gene set was mapped to a KEGG [56] (release 53) pathway to identify the best match classification for each gene. Finally, 72,293 protein coding genes (accounting for 99.30%) were functionally annotated.

Non-coding RNA annotation

The tRNA genes were predicted by tRNAscan-SE software (tRNAscan-SE, RRID:SCR_010835) [57]. The rRNA, miRNA and snRNA fragments were identified by INFERNAL [58] software (Infernal, RRID:SCR_011809) against the Rfam [59] database (release 9.1).

Gene family cluster

Gene families were generated using OrthoMCL [60]. First, nucleotide and protein data of 5 species (upland cotton, island cotton, wool cotton, yellow brown cotton and Darwin's cotton) were downloaded from the Ensembl (Release 70) and NCBI databases. Before an "all against all" BLASTP (E-value ≤ 1E-07) program, the longest transcript was selected from alternative splicing transcripts belonging to one gene and genes with ≤ 50 amino acids were then removed. Alignments with high-scoring *segment* pairs (HSPs) were conjoined for each gene pair using solar [61]. To identify homologous gene-pairs, a threshold of > 30% coverage of the aligned regions in both homologous genes was required. Finally, alignments were clustered into gene families using OrthoMCL utilizing a 1.5 inflation index. After clustering, 22,854 gene families were detected across Kobo&Cbra and four other species.

Phylogenetic tree construction and divergence time estimation

Single-copy orthologs were utilized to construct the phylogenetic tree. CDS sequences of these orthologs were aligned by MUSCLE (MUSCLE , RRID:SCR_011812) [62]. Using these CDS alignments, the phylogenetic tree was constructed by the ML (maximum likelihood) TREE algorithm in RAxML software [63, 64] (version 7.2.3, RRID:SCR_006086). Then the mcmctree program of PAML (PAML, RRID:SCR_014932) [65] was applied to estimate divergence time among 14 species with main parameters of burn-in=100,000, sample-number=100,000, and sample-frequency=2. Calibration points were selected and the TimeTree website was chosen as a normal prior to restrain the age of the nodes. The split of Kobo was estimated, close to that reported by others.

Gene family expansion and contraction

We determined the expansion and contraction of the gene families through comparing the cluster size differences between the ancestor and each species using the CAFÉ program [66]. A random birth and death model was used to study changes in gene families along each lineage of the phylogenetic tree. A probabilistic graphical model (PGM) was introduced to calculate the probability of transitions in gene family size from parent to child nodes in the phylogeny. Using conditional likelihoods as the test statistics, we calculated the corresponding *p*-values in each lineage. With a *p*-value of 0.05 used to identify gene families that were significantly expanded and contracted.

Screening of positively selected genes in CRI-12

The CDS alignments of single-copy gene families were generated using MUSCLE [62]. Gblocks

[66] was applied to filter poorly aligned positions and divergent regions of the CDS alignments. With Kobo and Rapi as foreground branches, positive selection sites were detected based on branch-site models of PAML [65] using CDS alignments. P values were computed using the χ^2 statistic and adjusted by the FDR method.

Whole-genome duplication analysis

We used BLASTP (E-value < 1e-5) to perform a homolog search with the Kobo genome and MCScanX was used to detect syntenic blocks. Then, Ks rates were calculated for all syntenic genes to identify putative whole genome duplication events in Kobo.

Whole-genome DNA methylation analysis

High-quality genomic DNA was isolated and used for the construction of DNA methylation library according to the previously described methods [67]. Methylation levels and differentially methylated regions (DMRs) were obtained using swDMR software [68]. Based on the results of haplotype block inheritance and recombination of agronomically important genes in CRI-12, conjoint analysis was performed to discover methylation haplotypes.

Abbreviations

Data availability

The sequencing data that support the findings of this study have been deposited in the CNGB Sequence Archive (CNSA) of China National GeneBank DataBase (CNGBdb) [69, 70] with accession number CNP0001942. In addition, the data used in the study is also available in the NCBI SRA under the BioProject number was PRJNA737739. All additional supporting data and materials are available in the *GigaScience* GigaDB database [71].

Abbreviations

bp: base pairs; SV: structural variations; PAV: presence/absence variations; WGBS: whole-genome bisulfite sequencing; MYA: million years ago; BUSCO: Benchmarking Universal Single-Copy Orthologs; Gb: gigabase pairs; kb: kilobase pairs; Mb: megabase pairs; 4DTv: 4-fold degenerate synonymous sites of the third codons; SDS: sodium dodecyl sulfate; SNP: single-nucleotide polymorphism; PE: paired-end; BWA: Burrows-Wheeler Aligner software; GATK: Genome Analysis Toolkit; PacBio: Pacific Biosciences; EPD: Eukaryotic Promoter Database; EVM: EVidenceModeler; GO: Gene Ontology; NCBI: National Center for Biotechnology Information; HSP: high-scoring segment pairs; PGM: probabilistic graphical model; DMR: differentially methylated regions; CNGBdb: China National GeneBank DataBase; MYA: million years ago.

Funding

This work was supported by the National Natural Science Foundation of China (32001460) and China Agriculture Research System of MOF and MARA.

Author’s contribution

WY and XL planned and designed the research. XL wrote the manuscript. XC, DW, ZY, JW, XF, SW, LG, LZ and RC performed experiments and analyzed the data. MD, CR, YF, YZ, LS, WM, MH and CC assist in revising papers and formats. XL, XC, DW, ZY, JW and XF contributed

equally. All authors have read and approved the manuscript.

Competing financial interests

The authors declare no competing financial interests.

Ethics approval and consent to participate

No ethical approval was required for this study.

Consent for publication

Not applicable.

Acknowledgements

We are grateful to Hangzhou LC-Bio Technology Co., Ltd for assisting in sequencing and bioinformatics analysis.

Additional files

Supplementary Figure S1: The certificate of the number one Chinese cotton variety CRI-12 and its breeder Mr. Tan Lianwang

Supplementary Figure S2: The percentage of different bases

Supplementary Figure S3: Phylogenetic and evolutionary analysis of the CRI-12

Supplementary Figure S4 Evidence support for the gene set

Supplementary Figure S5: Comparison of different elements in proximal species

Supplementary Figure S6: The distribution of the degree of ramification of TEs

Supplementary Figure S7: Motif features of CG-up methylation regions

Supplementary Table S1: Characters of CRI-12 and its parents

Supplementary Table S2: Statistics of sequencing data of *Gossypium hirsutum* L.

Supplementary Table S3: Details of CRI-12 genome assemblies

Supplementary Table S4: Statistics of genomic bases of *Gossypium hirsutum* L.

Supplementary Table S5: The number of clusters and length on each chromosome

Supplementary Table S6: BUSCO assessment results

Supplementary Table S7: Statistical results of gene functional annotation

Supplementary Table S8 statistics results of Repeat sequence

Supplementary Table S9: Classification Result Statistics of TEs

Supplementary Table S10: Details of non-coding RNAs in CRI-12 genome

Supplementary Table S11: Gene list by positive selection

Supplementary Table S12: Gene list in GO database

Supplementary Table S13: Gene list in KEGG database

Supplementary Table S14: Structural variations between different cotton species

Supplementary Table S15: PAV variations between different cotton species

Supplementary Table S16: Inherited SNP haplotypes were correlated with plant resistance in CRI-12

Supplementary Table S17: Statistics of haplotypes in different cotton species

Supplementary Table S18: DNA methylation haplotypes in CRI-12

References

1. Hu, Y, Chen J, Fang, L, et al. *Gossypium barbadense* and *Gossypium hirsutum* genomes

provide insights into the origin and evolution of allotetraploid cotton. Nat Genet 2019;51(4):739-48. doi:10.1038/s41588-019-0371-5.

2. Adams, KL, Wendel, JF. Polyploidy and genome evolution in plants. Current opinion in plant biology 2005;8(2):135-41. doi:10.1016/j.pbi.2005.01.001.

3. Paterson, AH, Wendel, JF, Gundlach, H, et al. Repeated polyploidization of *Gossypium* genomes and the evolution of spinnable cotton fibres. Nature 2012;492(7429):423-7. doi:10.1038/nature11798.

4. Wang, M, Tu, L, Lin, M, et al. Asymmetric subgenome selection and cis-regulatory divergence during cotton domestication. Nature genetics 2017;49(4):579-87. doi:10.1038/ng.3807.

5. Zhang, T, Hu, Y, Jiang, W, et al. Sequencing of allotetraploid cotton (*Gossypium hirsutum* L. acc. TM-1) provides a resource for fiber improvement. Nat Biotechnol 2015;33(5):531-7. doi:10.1038/nbt.3207.

6. Chen, ZJ, Scheffler, BE, Dennis, E, et al. Toward sequencing cotton (*Gossypium*) genomes. Plant Physiol 2007;145(4):1303-10. doi:10.1104/pp.107.107672.

7. Flagel, L, Udall, J, Nettleton, D, et al. Duplicate gene expression in allopolyploid *Gossypium* reveals two temporally distinct phases of expression evolution. BMC Biol 2008;6:16. doi:10.1186/1741-7007-6-16.

8. Pang, M, Woodward, AW, Agarwal, V, et al. Genome-wide analysis reveals rapid and dynamic changes in miRNA and siRNA sequence and expression during ovule and fiber development in allotetraploid cotton (*Gossypium hirsutum* L.). Genome Biol 2009;10(11):R122. doi:10.1186/gb-2009-10-11-r122.

9. Li, F, Fan, G, Lu, C, et al. Genome sequence of cultivated Upland cotton (*Gossypium hirsutum* TM-1) provides insights into genome evolution. Nat Biotechnol 2015;33(5):524-30. doi:10.1038/nbt.3208.

10. Wang, M, Tu, L, Yuan, D, et al. Reference genome sequences of two cultivated allotetraploid cottons, *Gossypium hirsutum* and *Gossypium barbadense*. Nature Genetics 2019;51(2):224-9. doi:10.1038/s41588-018-0282-x.

11. Yang, Z, Ge, X, Yang, Z, et al. Extensive intraspecific gene order and gene structural variations in upland cotton cultivars. Nat Commun 2019;10(1):2989. doi:10.1038/s41467-019-10820-x.

12. Lu, X, Fu, X, Wang, D, et al. Resequencing of cv CRI-12 family reveals haplotype block inheritance and recombination of agronomically important genes in artificial selection. Plant Biotechnol J 2019;17(5):945-55. doi:10.1111/pbi.13030.

13. Du, X, Huang, G, He, S, et al. Resequencing of 243 diploid cotton accessions based on an updated A genome identifies the genetic basis of key agronomic traits. Nat Genet 2018;50(6):796-802. doi:10.1038/s41588-018-0116-x.

14. Edger, PP, Poorten, TJ, VanBuren, R, et al. Origin and evolution of the octoploid strawberry genome. Nat Genet 2019;51(3):541-7. doi:10.1038/s41588-019-0356-4.

15. Raymond, O, Gouzy, J, Just, J, et al. The Rosa genome provides new insights into the domestication of modern roses. Nature Genetics 2018;50(6):772-777. doi:10.1038/s41588-018-0110-3.

16. Feschotte, C, Jiang, N, Wessler, SR. Plant transposable elements: where genetics meets genomics. Nature reviews Genetics 2002;3(5):329-41. doi:10.1038/nrg793.

17. Ullah A, Qamar, MT, Nisar, M, et al. Characterization of a novel cotton MYB gene, GhMYB108-like responsive to abiotic stresses. Mol Biol Rep 2020;47(3):1573-81.

doi:10.1007/s11033-020-05244-6.
18. Pottier, MA, Bozzolan, F, Chertemps, T, et al. Cytochrome P450s and cytochrome P450 reductase in the olfactory organ of the cotton leafworm Spodoptera littoralis. Insect molecular biology 2012;21(6):568-80. doi:10.1111/j.1365-2583.2012.01160.x.
19. Lu, X, Shu, N, Wang, D, et al. Genome-wide identification and expression analysis of PUB genes in cotton. BMC genomics 2020;21(1):213. doi:10.1186/s12864-020-6638-5.
20. Li F, Fan G, Wang K, et al. Genome sequence of the cultivated cotton *Gossypium arboreum*. Nat Genet 2014;46(6):567-72. doi:10.1038/ng.2987.
21. Kawakatsu, T, Huang, SC, Jupe, F, et al. Epigenomic Diversity in a Global Collection of *Arabidopsis thaliana* Accessions. Cell 2016;166(2):492-505. doi:10.1016/j.cell.2016.06.044.
22. Sahu, SK, Thangaraj, M, Kathiresan, K. DNA Extraction Protocol for Plants with High Levels of Secondary Metabolites and Polysaccharides without Using Liquid Nitrogen and Phenol. ISRN Mol Biol 2012;2012:205049. doi:10.5402/2012/205049.
23. Chen, N. Using RepeatMasker to identify repetitive elements in genomic sequences. Current protocols in bioinformatics 2004;Chapter 4:Unit 4 10. doi:10.1002/0471250953.bi0410s05.
24. Price, AL, Jones, NC and Pevzner, PA. De novo identification of repeat families in large genomes. Bioinformatics 2005;21:I351-I8. doi:10.1093/bioinformatics/bti1018.
25. Xu, Z, Wang, H. LTR_FINDER: an efficient tool for the prediction of full-length LTR retrotransposons. Nucleic Acids Res 2007;35:W265-W8. doi:10.1093/nar/gkm286.
26. Benson, G. Tandem repeats finder: a program to analyze DNA sequences. Nucleic Acids Res 1999;27(2):573-80. doi:DOI 10.1093/nar/27.2.573.
27. Belton, JM, McCord, RP, Gibcus, JH, et al. Hi-C: a comprehensive technique to capture the conformation of genomes. Methods 2012;58(3):268-76. doi:10.1016/j.ymeth.2012.05.001.
28. Li, H, Durbin, R. Fast and accurate short read alignment with Burrows-Wheeler transform. Bioinformatics 2009;25(14):1754-60. doi:10.1093/bioinformatics/btp324.
29. Danecek P, Bonfield JK, Liddle J, Marshall J, Ohan V, Pollard MO, Whitwham A, Keane T, McCarthy SA, Davies RM, Li H. Twelve years of SAMtools and BCFtools. Gigascience. 2021 Feb 16;10(2):giab008. doi: 10.1093/gigascience/giab008.
30. McKenna, A, Hanna, M, Banks, E, et al. The Genome Analysis Toolkit: A MapReduce framework for analyzing next-generation DNA sequencing data. Genome Res 2010;20(9):1297-303. doi:10.1101/gr.107524.110.
31. Selvaraj, S, Dixon, JR, Bansal, V, et al. Whole-genome haplotype reconstruction using proximity-ligation and shotgun sequencing. Nature Biotechnology 2013;31(12):1111-1118. doi:10.1038/nbt.2728.
32. Wingett, S, Ewels, P, Furlan-Magaril, M, et al. HiCUP: pipeline for mapping and processing Hi-C data. 2015;4:1310. doi:10.12688/f1000research.7334.1.
33. Langmead, B, Salzberg, SL. Fast gapped-read alignment with Bowtie 2. Nat Methods 2012;9(4):357-9. doi:10.1038/nmeth.1923.
34. Yaffe, E, Tanay, A. Probabilistic modeling of Hi-C contact maps eliminates systematic biases to characterize global chromosomal architecture. Nature genetics 2011;43(11):1059-65. doi:10.1038/ng.947.
35. Kim, D, Langmead, B, Salzberg, SL. HISAT: a fast spliced aligner with low memory requirements. Nature methods 2015;12(4):357-60. doi:10.1038/nmeth.3317. doi:10.1038/nmeth.3317.

36. Pertea, M, Pertea, GM, Antonescu, CM, et al. StringTie enables improved reconstruction of a transcriptome from RNA-seq reads. Nature biotechnology 2015;33(3):290-5. doi:10.1038/nbt.3122.
37. Robinson, MD, McCarthy, DJ, Smyth, GK. edgeR: a Bioconductor package for differential expression analysis of digital gene expression data. Bioinformatics 2010;26(1):139-40. doi:10.1093/bioinformatics/btp616.
38. Kanehisa, M, Araki, M, Goto, S, et al. KEGG for linking genomes to life and the environment. Nucleic Acids Res 2008;36(Database issue):D480-4. doi:10.1093/nar/gkm882.
39. Young, MD, Wakefield, MJ, Smyth, GK, et al. Gene ontology analysis for RNA-seq: accounting for selection bias. Genome Biol 2010;11(2):R14. doi:10.1186/gb-2010-11-2-r14.
40. Stanke, M, Schoffmann, O, Morgenstern, B, et al. Gene prediction in eukaryotes with a generalized hidden Markov model that uses hints from external sources. BMC Bioinformatics 2006;7:62. doi:10.1186/1471-2105-7-62.
41. Stanke, M, Waack, S. Gene prediction with a hidden Markov model and a new intron submodel. Bioinformatics 2003;19(Suppl 2):ii215-25. doi:10.1093/bioinformatics/btg1080.
42. Salamov, AA, Solovyev, VV. Ab initio gene finding in Drosophila genomic DNA. Genome research 2000;10(4):516-22. doi:Doi 10.1101/Gr.10.4.516.
43. Parra, G, Blanco, E, Guigo, R. GeneID in Drosophila. Genome research 2000;10(4):511-5. doi:Doi 10.1101/Gr.10.4.511.
44. Majoros, WH, Pertea, M, Salzberg, SL. TigrScan and GlimmerHMM: two open source ab initio eukaryotic gene-finders. Bioinformatics 2004;20(16):2878-9. doi:10.1093/bioinformatics/bth315.
45. Korf, I. Gene finding in novel genomes. BMC Bioinformatics 2004;5:59. doi:10.1186/1471-2105-5-59.
46. Altschul, SF, Madden, TL, Schaffer, AA, et al. Gapped BLAST and PSI-BLAST: a new generation of protein database search programs. Nucleic Acids Res 1997;25(17):3389-402. doi:10.1093/nar/25.17.3389.
47. Birney, E, Clamp, M, Durbin, R. GeneWise and genomewise. Genome research 2004;14(5):988-95. doi:10.1101/gr.1865504.
48. Trapnell, C, Pachter, L, Salzberg, SL. TopHat: discovering splice junctions with RNA-Seq. Bioinformatics 2009;25(9):1105-11. doi:10.1093/bioinformatics/btp120.
49. Trapnell, C, Williams, BA, Pertea, G, et al. Transcript assembly and quantification by RNA-Seq reveals unannotated transcripts and isoform switching during cell differentiation. Nature biotechnology 2010;28(5):511-U174. doi:10.1038/nbt.1621.
50. Grabherr, MG, Haas, BJ, Yassour, M, et al. Full-length transcriptome assembly from RNA-Seq data without a reference genome. Nat Biotechnol 2011;29(7):644-52. doi:10.1038/nbt.1883.
51. Haas, BJ, Delcher, AL, Mount, SM, et al. Improving the Arabidopsis genome annotation using maximal transcript alignment assemblies. Nucleic Acids Res 2003;31(19):5654-66. doi:10.1093/nar/gkg770.
52. Haas, BJ, Salzberg, SL, Zhu, W, et al. Automated eukaryotic gene structure annotation using EVidenceModeler and the program to assemble spliced alignments. Genome Biology 2008;9(1): doi:ARTN R710.1186/gb-2008-9-1-r7. doi:ARTN R710.1186/gb-2008-9-1-r7.
53. Bairoch, A, Apweiler, R. The SWISS-PROT protein sequence database and its supplement TrEMBL in 2000. Nucleic acids research 2000;28(1):45-8. doi:10.1093/nar/28.1.45.

54. Mulder, N, Apweiler, R. InterPro and InterProScan: tools for protein sequence classification and comparison. Methods Mol Biol 2007;396:59-70. doi:10.1007/978-1-59745-515-2_5.
55. Ashburner, M, Ball, CA, Blake, JA, et al. Gene ontology: tool for the unification of biology. The Gene Ontology Consortium. Nature genetics 2000;25(1):25-9. doi:10.1038/75556.
56. Kanehisa, M, Goto, S. KEGG: kyoto encyclopedia of genes and genomes. Nucleic acids research 2000;28(1):27-30. doi:10.1093/nar/28.1.27.
57. Lowe, TM, Eddy, SR. tRNAscan-SE: a program for improved detection of transfer RNA genes in genomic sequence. Nucleic acids research 1997;25(5):955-64. doi:10.1093/nar/25.5.955.
58. Nawrocki, EP, Kolbe, DL, Eddy, SR. Infernal 1.0: inference of RNA alignments. Bioinformatics 2009;25(10):1335-7. doi:10.1093/bioinformatics/btp157.
59. Griffiths-Jones, S, Moxon, S, Marshall, M, et al. Rfam: annotating non-coding RNAs in complete genomes. Nucleic acids research 2005;33(Database issue):D121-4. doi:10.1093/nar/gki081.
60. Li, L, Stoeckert, CJ, Roos, DS. OrthoMCL: identification of ortholog groups for eukaryotic genomes. Genome research 2003;13(9):2178-89. doi:10.1101/gr.1224503.
61. Yu, XJ, Zheng, HK, Wang, J, et al. Detecting lineage-specific adaptive evolution of brain-expressed genes in human using rhesus macaque as outgroup. Genomics 2006;88(6):745-51. doi:10.1016/j.ygeno.2006.05.008.
62. Edgar, RC. MUSCLE: multiple sequence alignment with high accuracy and high throughput. Nucleic Acids Res 2004;32(5):1792-7. doi:10.1093/nar/gkh340.
63. Stamatakis, A. RAxML-VI-HPC: maximum likelihood-based phylogenetic analyses with thousands of taxa and mixed models. Bioinformatics 2006;22(21):2688-90. doi:10.1093/bioinformatics/btl446.
64. Stamatakis, A, Hoover, P, Rougemont, J. A rapid bootstrap algorithm for the RAxML Web servers. Systematic biology 2008;57(5):758-71. doi:10.1080/10635150802429642.
65. Yang, Z. PAML 4: phylogenetic analysis by maximum likelihood. Mol Biol Evol 2007;24(8):1586-91. doi:10.1093/molbev/msm088.
66. Zhang, J, Nielsen, R, Yang, Z. Evaluation of an improved branch-site likelihood method for detecting positive selection at the molecular level. Mol Biol Evol 2005;22(12):2472-9. doi:10.1093/molbev/msi237.
67. Lu, X, Wang, X, Chen, X, et al. Single-base resolution methylomes of upland cotton (*Gossypium hirsutum* L.) reveal epigenome modifications in response to drought stress. BMC genomics 2017;18(1):297. doi:10.1186/s12864-017-3681-y.
68. Wang Z, Li X, Jiang Y, Shao Q, Liu Q, Chen B, Huang D. swDMR: A Sliding Window Approach to Identify Differentially Methylated Regions Based on Whole Genome Bisulfite Sequencing. PLoS One. 2015 Jul 15;10(7):e0132866. doi: 10.1371/journal.pone.0132866.
69. Chen, FZ, You, LJ, Yang, F, et al. CNGBdb: China National GeneBank DataBase. Hereditas 2020;42(8):799-809. doi:10.16288/j.yczz.20-080.
70. Guo, X, Chen, F, Gao, F, et al. CNSA: a data repository for archiving omics data. Database (Oxford) 2020: doi:10.1093/database/baaa055.
71. Lu, X, Chen, X, Wang, D, et al. Supporting data for "A high-quality assembled genome and its comparative analysis decode the adaptive molecular mechanism of the number one Chinese cotton variety CRI-12" GigaScience Database 2022. http://dx.doi.org/10.5524/100984.

Figures

Figure 1: Phenotype of CRI-12 and synteny relation of different cotton genomes

a, Phenotype of CRI-12. b, Hi-C map of CRI-12. From left to right, each square represents a chromosome from chromosome 1 to 26. c, Synteny analysis of different cotton species. d, Whole-genome duplications in Malvales through 4DTv analysis. The percentages of the orthologous gene pairs between CRI-12 and other species and 4DTv values were calculated. Fourfold Degenerate Synonymous Site (4DTV) is used as an evolutionary parameter to evaluate whether a genome-wide replication event occurs. A site of a codon at which all nucleotides encode the same amino acid is a quadruple degenerate site.

Figure 2: Genomic landscape of CRI-12 genome

From the outside to the inside, each circle represents LTR retrotransposons density, LINE retrotransposons density, SINE retrotransposons density, DNA transposons, gene density, GC content, miRNA density in 1Mb sliding windows.

Figure 3: Genome annotation of CRI-12

a, Statistical results of gene functional annotation. b, Number of common and unique gene families. c, Expansion and contraction in gene families. c, Expression analysis of partial positive selection genes under *V. wilt*, salt and drought stress.

Figure 4: Statistics of pathway enrichment of SV- and PAV- correlated genes

a, GO enrichment of SV variations related genes. Gene Ontology (GO) contains three components, cellular component (CC), molecular function (MF), and biological process (BP). Pink bars represent MF, green bars represent CC, and orange bars represent BP. b, GO enrichment of PAV variations related genes. Purple bars represent MF, orange bars represent CC, and green bars represent BP. Only several main enrichments were listed. c, Statistics of pathway enrichment of SV variations related genes. d, Statistics of pathway enrichment of PAV variations related genes.

Figure 5: Haplotypes in polyploidization of diploid cottons

Red bars indicate haplotypes, more red bars, higher the haplotype density. A01 to A13 represent thirteen chromosomes in A genome (*Gossypium arboreum*), while D01 to D13 represent thirteen chromosomes in D genome (*Gossypium raimondii*). At and Dt represent A subgenome and D subgenome in *Gossypium hirsutum* L., and Chr01 to Chr13 represent thirteen chromosomes in each subgenome. Tetraploid cottons *G. hirsutum* L. and *G. barbadense* L. originated from the hybridization of A-genome-like ancestors and D-genome-like ancestors about 1-1.5 million years ago (MYA).

Figure 6: Methylation Haplotypes in CRI-12 provide adaptive advantages to abiotic stresses

CRI-12 was developed from a cross between Xingtai6871 (♂) and Uganda4 (♀) in disease nursery and serious illness field. Methylation haplotypes in two parents could be inherited to CRI-12. A total of 66 methylation haplotypes were discovered in CRI-12, 8 of which were located on chromosome D13. Methylation haplotypes in CRI-12 could provide adaptive advantages to

different stresses.

Tables

Table 1 Global statistical analysis of CRI-12

Category	CRI-12
Total contig (Mb)	2,199.32
Contig N50 (Mb)	19.65
Total scaffold N50 (Mb)	2,199.32
Scaffold N50 (Mb)	91.74
Scaffold L50 (Mb)	108
Longest scaffold (Mb)	127.37
Gene anchored and oriented (%)	98.55
Gap Size (bp)	59,500
Fragmented	1.2%
Missing	1.6%
miRNAs (Mb) (copy)	523
tRNAs (copy)	2,214
rRNAs (copy)	5,498
snRNAs (copy)	16,320
Repeat sequence (%)	63.55
TE proportion (%)	62.57

Note: Contigs with longer than 100bp were selected for the genome assembly.

Table 2 Prediction results of gene structure

Gene set		Number	Average gene length (bp)	Average CDS length (bp)	Average exons per gene	Average exon length (bp)	Average intron length (bp)
De novo	Augustus	87,013	2,286.03	1,015.76	4.38	232.12	376.26
	GlimmerHMM	179,486	10,803.61	568.18	3.01	188.95	5,099.91
	SNAP	129,571	3,395.43	550.58	3.59	153.49	1,099.60
	Geneid	139,759	4,699.24	682.85	3.92	174.26	1,376.15
	Genscan	97,875	13,613.92	970.22	5.17	187.61	3,030.99
Homolog	Ath	145,589	1,307.79	705.31	2.57	274.02	382.79
	Gar	86,180	2,585.62	1,194.63	3.88	308.10	483.43
	Gba	76,449	2,409.54	1,223.53	3.90	313.74	408.99
	Ghi_L	319,648	1,122.67	630.78	2.16	292.64	425.70
	Ghi_ZM24	250,369	2,726.79	1,186.63	3.69	321.53	531.69
	Gra	78,710	2,679.26	1,352.37	4.61	293.11	443.88
RNA-seq	PASA	117,481	2,387.37	911.22	4.31	211.36	445.80
	Cufflinks	114,567	4,007.92	1,770.99	5.66	313.03	480.28
EVM		97,655	2,415.39	951.18	4.23	224.92	453.45
Pasa-update*		97,360	2,690.87	945.51	4.16	227.12	464.73
Final set*		72,293	2,834.36	1,134.46	4.98	227.97	427.50

Note: Denovo prediction of gene structure was performed with softwares Augustus, GlimmerHMM, SNAP, Geneid and Genscan；Species annotated contained *Arabidopsis thaliana*, *Gossypium arboreum*, *Gossypium barbadense*, *Gossypium hirsutum* L., *Gossypium hirsutum* ZM24 and *Gossypium raimondii*.

Click here to access/download
Supplementary Material
Supplemental tables.xlsx

Supplementary figures

Click here to access/download
Supplementary Material
Supplemental figures.pdf
