# Supplementary material for: A high-quality assembled genome and its comparative analysis decode the adaptive molecular mechanism of the number one Chinese cotton variety CRI-12
Source: Gigascience. 2022 Apr 1;11:giac019. doi: 10.1093/gigascience/giac019 (PMC8975723; doi:10.1093/gigascience/giac019)
Supplement: giac019_Supplemental_Figures_and_Tables [file giac019_supplemental_figures_and_tables.zip › Supplemental figures.pdf]

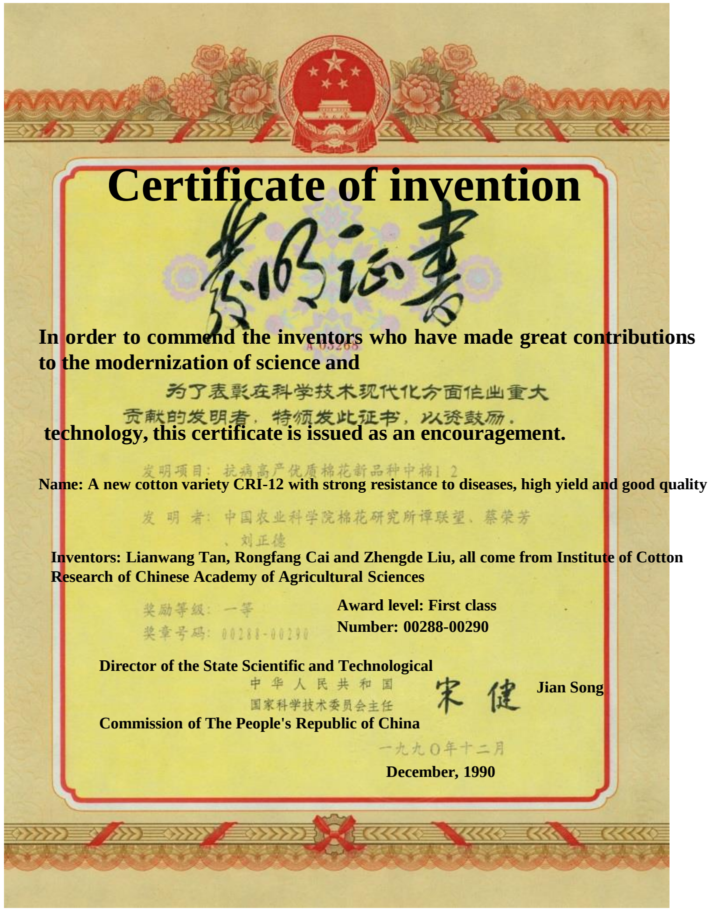

a

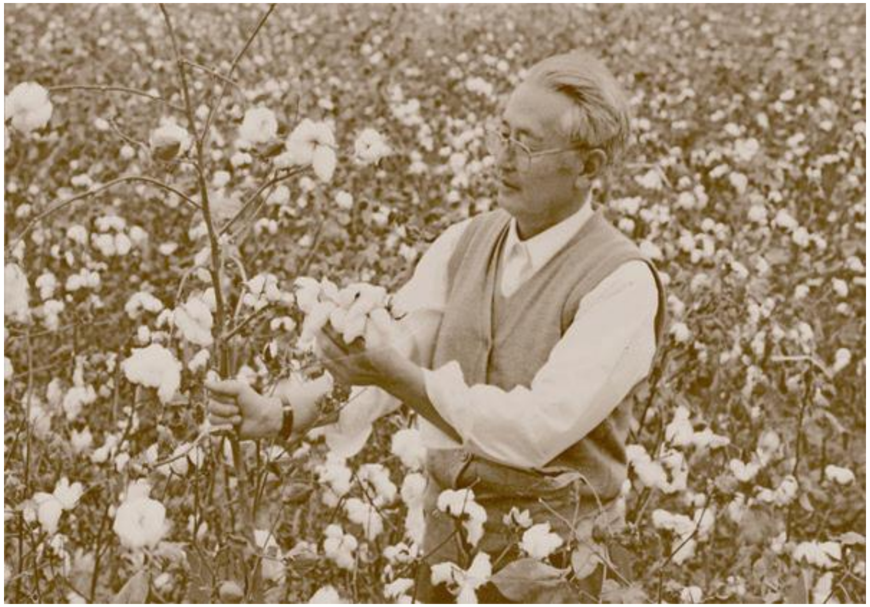

b

Figure S1. The certificate of the number one Chinese cotton variety CRI-12 and its breeder Mr. Tan Lianwang

a represents the certificate of the number one Chinese cotton variety CRI-12. b represents the famous cotton breeder Mr. Tan Lianwang, one of the major breeders of CRI-12.

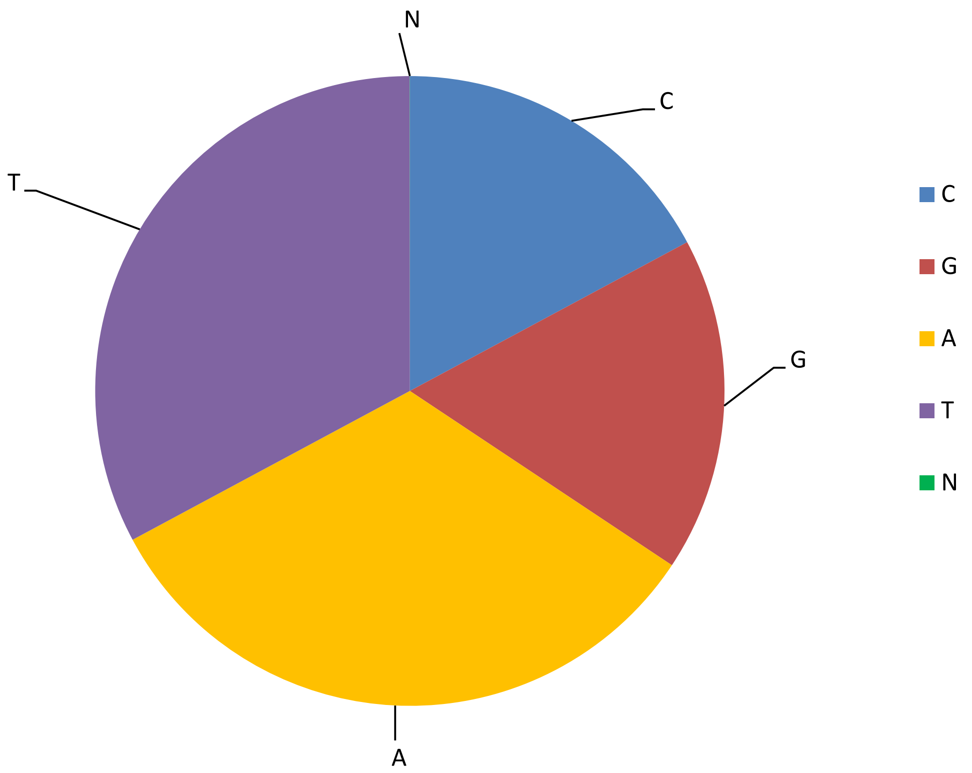

Figure S2. The percentage of different bases

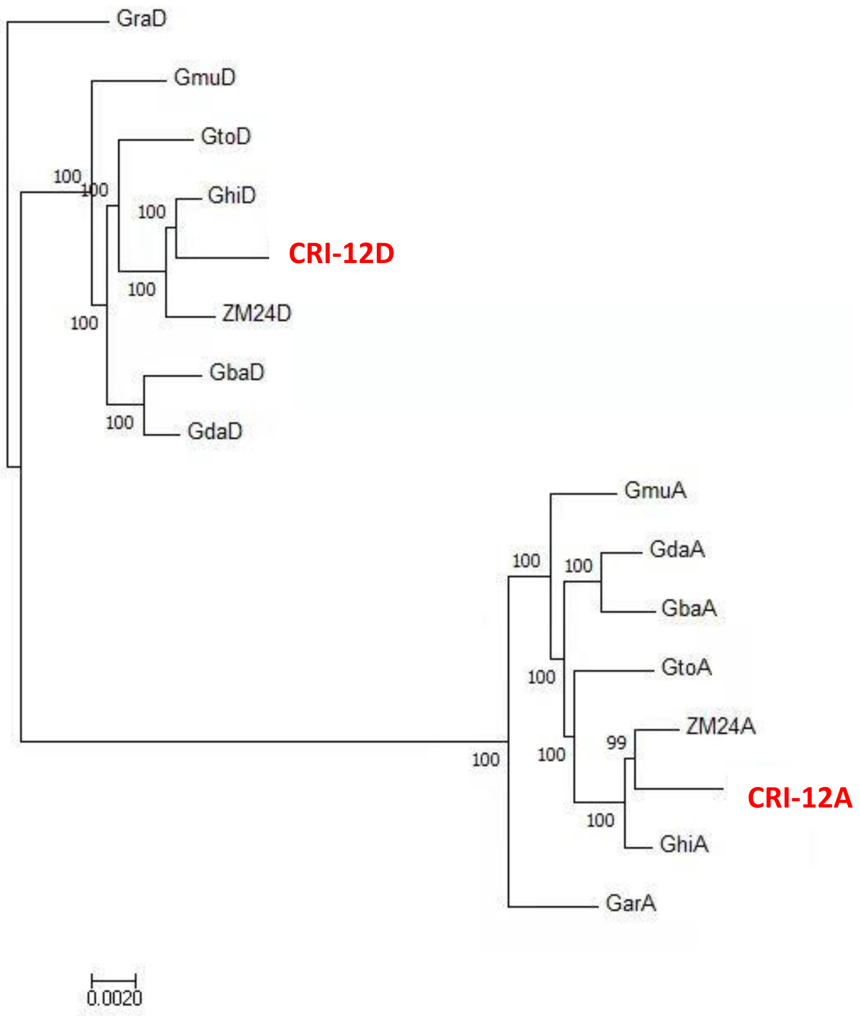

Figure S3. Phylogenetic and evolutionary analysis of the CRI-12

Note: Gra represents *Gossypium raimondii*; Gmu *represents Gossypium mustelinum*; Gto represents *Gossypium tomentosum*; Ghi represents *Gossypium hirsutum* L TM-1.; ZM24 represents *Gossypium hirsutum* zhongmiansuo24; Gba represents *Gossypium barbadense*; Gda represents *Gossypium darwinii*.

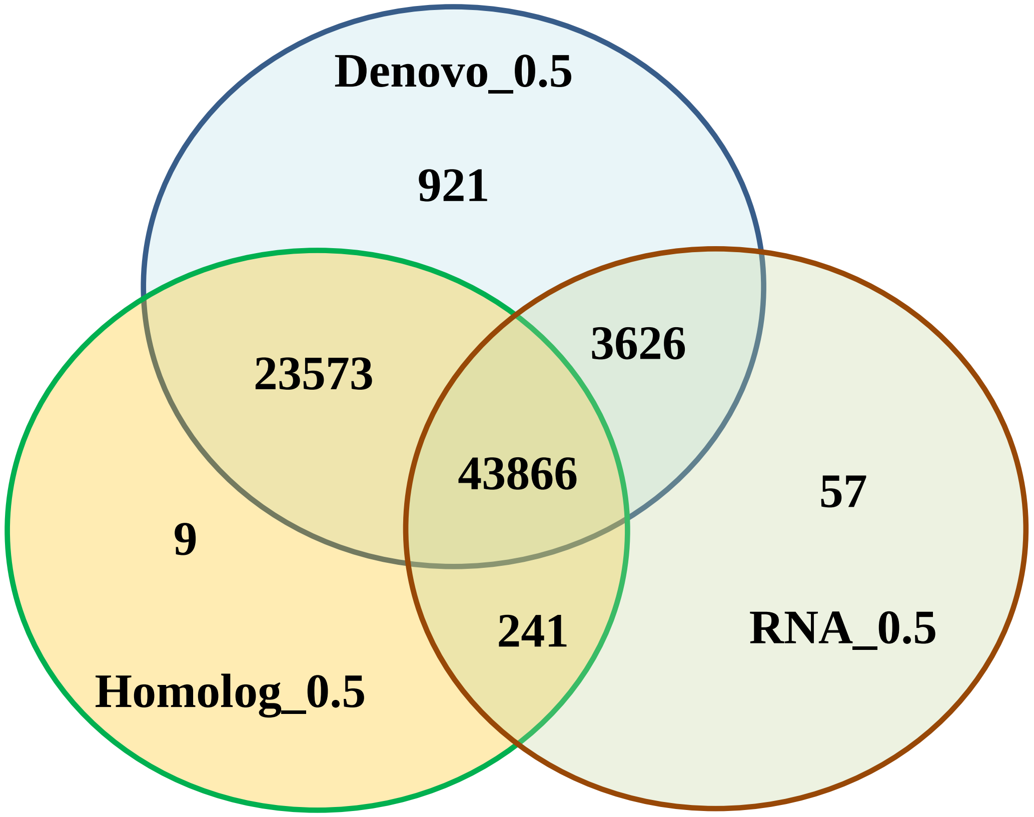

Figure S4. Evidence support for the gene set

Note: Denovo, Homolog and RNA showed predicted genes supported by denovo, homolog and RNA-seq, respectively. 0.5 means that the gene overlap was above 50%.

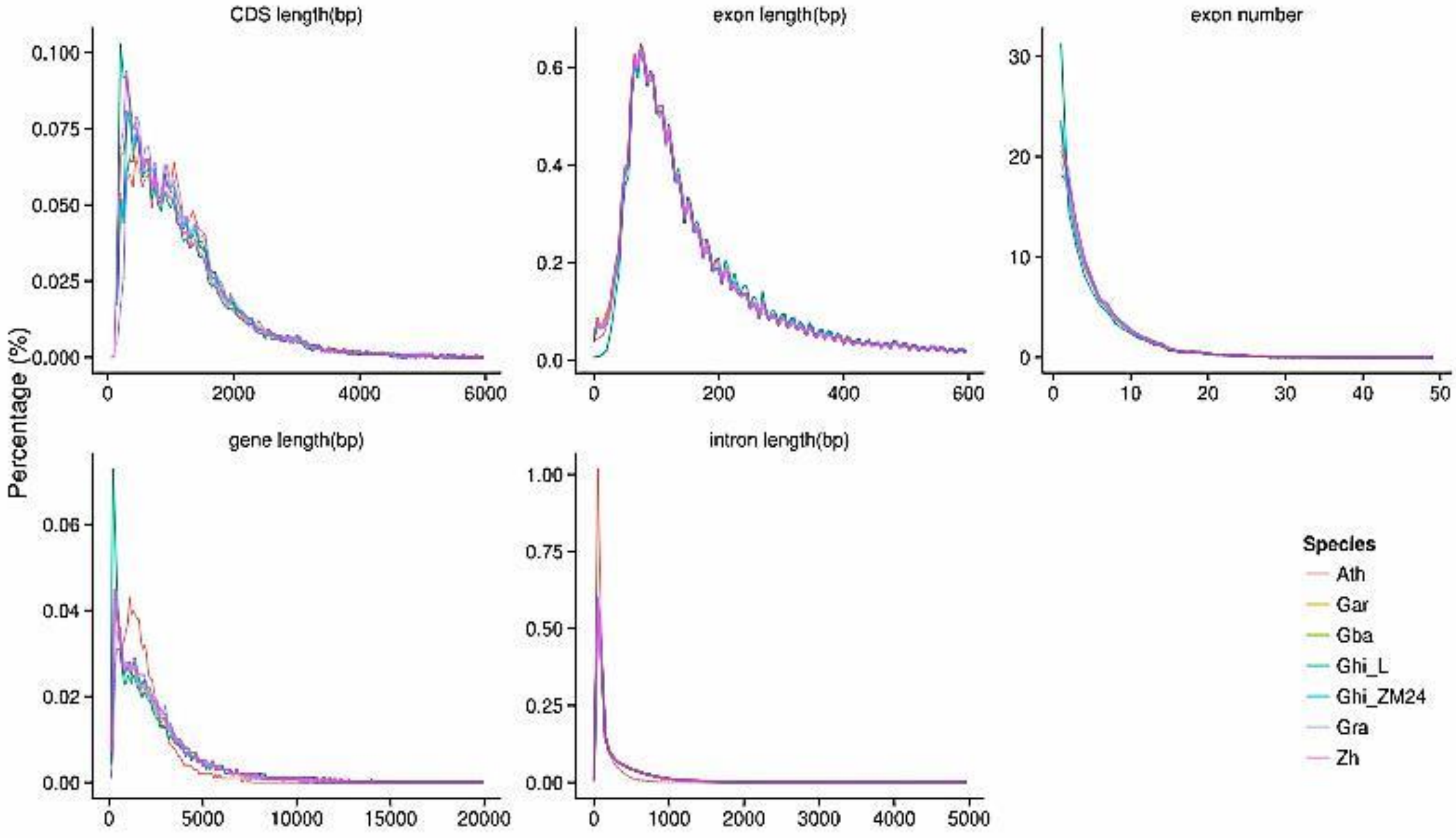

Figure S5. Comparison of different elements in proximal species

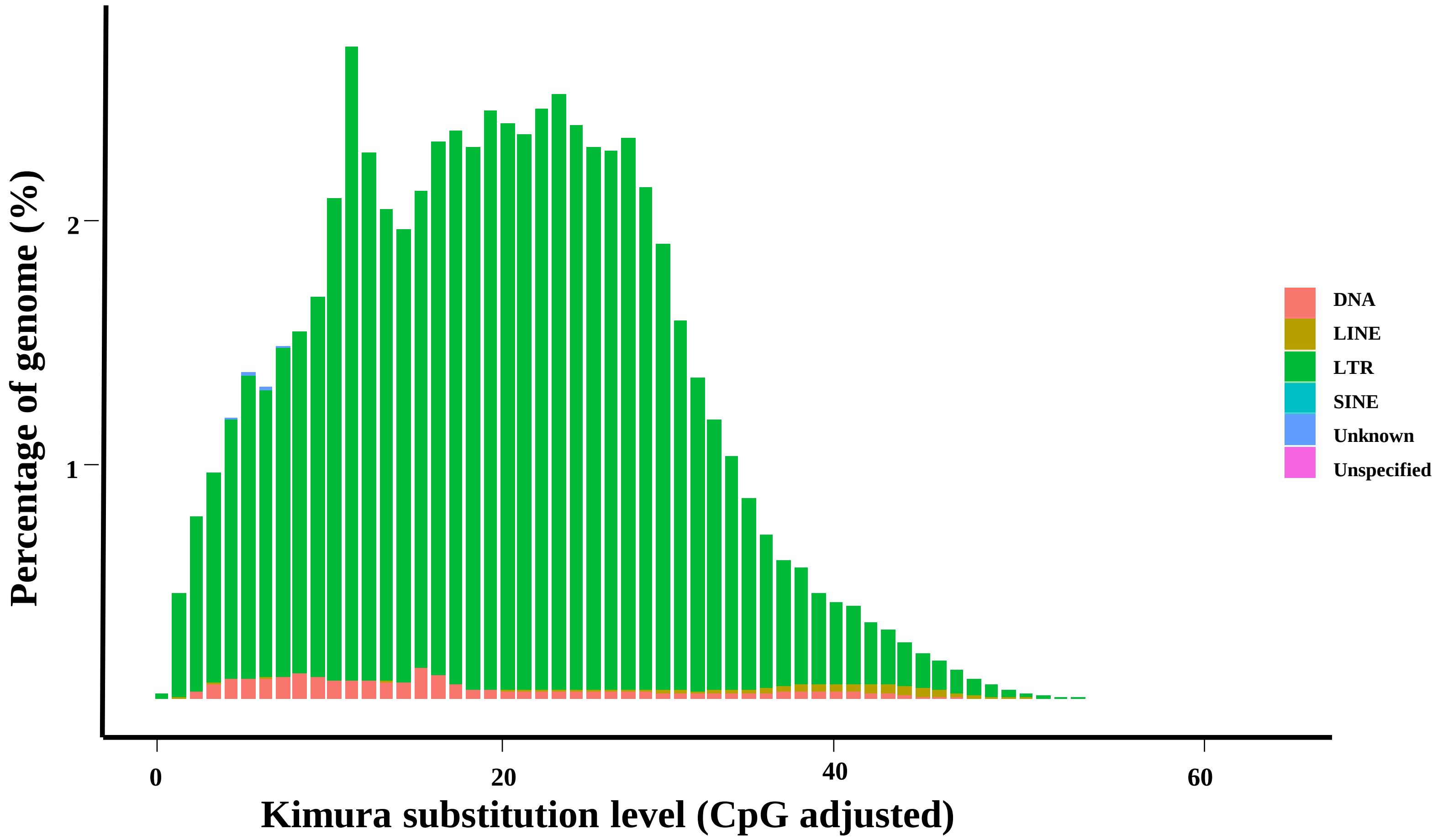

Figure S6. The distribution of the degree of ramification of TEs

Note: X axis represents the degree of ramification of TEs and Y axis represents the percentage of TEs in the genome at each degree of ramification. Different colors indicated different TEs.

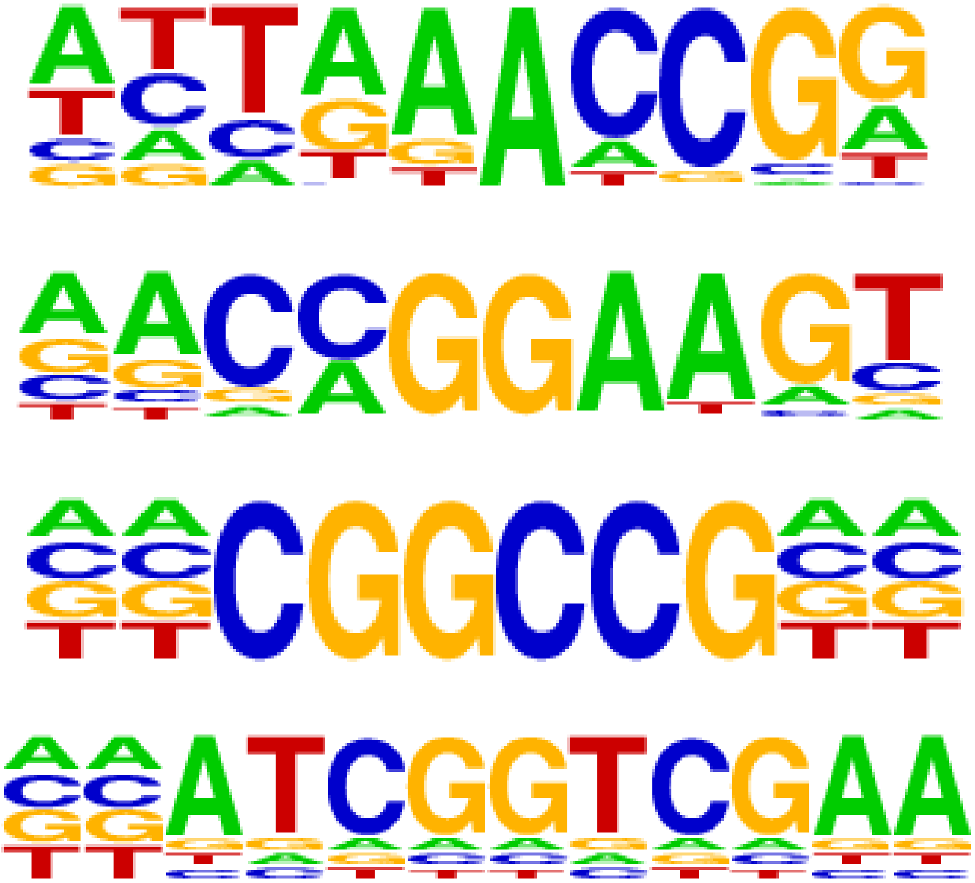

Figure S7 . Motif features of CG-up methylation regions
