# Supplementary material for: A high-quality assembled genome and its comparative analysis decode the adaptive molecular mechanism of the number one Chinese cotton variety CRI-12
Source: Gigascience. 2022 Apr 1;11:giac019. doi: 10.1093/gigascience/giac019 (PMC8975723; doi:10.1093/gigascience/giac019)
Supplement: giac019_Response_to_Reviewer_Comments_Revision_1 [file giac019_response_to_reviewer_comments_revision_1.pdf]

Author's Response To Reviewer Comments

Close

Dear editor and two reviewers,
Thanks very much for your patience for processing our manuscript entitled “A high-quality assembled genome and its comparative analysis decode the adaptive molecular mechanism of Chinese No.1 cotton variety CRI-12” (GIGA-D-21-00277). We are also very grateful for the two reviewers’ hard work on reviewing our manuscript and put forward these valuable suggestions. Now we have revised the manuscript as the comments one by one. The details could be found be below.
Reviewer 1:
Q1: Assembly: The authors used Pacbio + HiC to generate a high-quality genome assembly. Although they described the details regarding Pacbio data assembly. I couldn't find much details regarding how the HiC scaffolding was performed
Answer: Thanks for the suggestion. In the manuscript, We have added the details regarding how the Hi-C scaffolding was performed.

Q2: The authors used different methods to evaluate the quality of the assembled genomes and obtained good results. Although the BUSCO score is good. It is better to use additional software to evaluate this genome quality. e.g., LTR Assembly Index (LAI), an important criterion for the genome quality nowadays, particularly for genome with a high percentage of TEs.
Answer: It is a valuable suggestion. We re-assess the CRI-12 genome with the LTR Assembly Index (LAI) analysis in the manuscript and the results showed that the newly assembled CRI-12 genome reached the level of reference genome.

Q3: Fig. 1b and 1d. The font size is too small to read the content.
Answer: We have revised the font size of the Fig 1b and 1d.

Q4: DNA extraction method lack reference citation.
Answer: We have added a reference citation for DNA extraction.

Q5: Several methods are presented in too much details. If the original references are there, the only brief descriptions would be enough with relevant citations.
Answer：It was a good idea. We have simplified several methods, e.g. PacBio sequencing and Hi-C read mapping and filtering and generation of contact matrices.

Q6: Figure S1a. The content of the certificate should be translated in English and described in legends for non-chinese readers.
Answer: This was a very good suggestion. We have added English legends on the figure S1a.

Q7: I was wondering if there is any full name of CRI? If yes, please describe in Introduction section
Answer: Thanks for your advice. We have added full name of CRI-12 in introduction section for the first appearance.

Q8: Line 578. Check the spelling/grammar and rewrite the sentence "And the remain data…"
Answer: We have revised the sentence in the manuscript using “And the rest of data …”.

Q9: Check the image quality of the Supplementary figures.
Answer: We have revised the supplementary figures.

Reviewer 2:
Q1: The manuscript would significantly benefit if the Author removes repeated sentences throughout the manuscript (e.g. lines 113-114 and 123-124 or Lines 81-82 and 85-86).
Answer: Thanks for your valuable suggestion. We have revised the repeated descriptions throughout the manuscript.

Q2: The authors described results related to methylation and SVs like CNVs or PVA, but their

descriptions are not in Material and methods.
Answer: The descriptions related to methylation and SVs were added in the “Materials and method” section.

Q3: Please add the number of biological replicates.
Answer: We have added the number of biological replicates in the materials and method section.

Q4: The discussion is like a summary; therefore, I would suggest adding a conclusion instead.
Answer: Thanks for your suggestion. We added a conclusion in the manuscript.

Q5: Lines 54-59: Please modify and add as a new paragraph.
Answer: We have modified and added a new paragraph.

Q6: - LineS 77-78: Please modify for clarification.
Answer: We have revised the description.

Q7:- Line 84: Please remove "finally" and modify the sentence.
Answer: We removed “finally” and replaced it with “In total”.

Q8:- Lines 254-256: Please modify for clarification.
Answer: We have modified the sentence for clarification.

Q9:- Line 267: "… two high-quality…" could you please add the name
Answer: We have added the name of two high-quality genomes in the manuscript.

Q10:- Line 521: Could you please add the name of 14 species.
Answer: We have revised the description of this sentence.

Q11:- Please clarify if "figure 3c" is from previous or current work.
Answer: In current work, we performed the analysis of expanded and contracted gene families related to stress tolerance in CRI-12. So we confirmed it that Figure 3c is from our current work.

Q12:- Line 548: Please add the full name for "PTE"?
Answer: We have revised the title in Line 548.

Q13: - Please spell out all abbreviations and botanical names for the first time in the main text; after that, you can use the abbreviation and the short term.
Answer: We have checked all abbreviations and botanical names and spelt out the full names for the first time.

Q14:- Latin name should be written in italics.
Answer: We have rechecked the latin names in the manuscript and revised them in italics.

Many thanks again for your patience and consideration.

Close
