# Supplementary material for: A high-quality assembled genome and its comparative analysis decode the adaptive molecular mechanism of the number one Chinese cotton variety CRI-12
Source: Gigascience. 2022 Apr 1;11:giac019. doi: 10.1093/gigascience/giac019 (PMC8975723; doi:10.1093/gigascience/giac019)
Supplement: giac019_Response_to_Reviewer_Comments_Revision_2 [file giac019_response_to_reviewer_comments_revision_2.pdf]

Author's Response To Reviewer Comments

Close

Dear editor and two reviewers,
Thanks very much for your patience for processing our manuscript entitled “A high-quality assembled genome and its comparative analysis decode the adaptive molecular mechanism of Chinese No.1 cotton variety CRI-12” (GIGA-D-21-00277). We are also very grateful for the two reviewers’ hard work on reviewing our manuscript and put forward these valuable suggestions. Now we have revised the manuscript as the comments one by one. The details could be found be below.
Reviewer 1:
Q1: The authors have nicely addressed all the raised concerns. But, the cited reference for DNA extraction, doesn't seem to be a correct one. Please check,
and the relevant DNA extraction methods such as Sahu et al, 2012
(https://pubmed.ncbi.nlm.nih.gov/27335662/) or any other suitable method or kits.
Answer: Thanks for the suggestion. We have replaced the reference for DNA extraction with the recommended citation in the manuscript.

Many thanks again for your patience and consideration.

Close
