# Supplementary material for: A high-quality assembled genome and its comparative analysis decode the adaptive molecular mechanism of the number one Chinese cotton variety CRI-12
Source: Gigascience. 2022 Apr 1;11:giac019. doi: 10.1093/gigascience/giac019 (PMC8975723; doi:10.1093/gigascience/giac019)
Supplement: giac019_Reviewer_1_Report_Original_Submission — Sunil Kumar Sahu, PhD., -- 11/18/2021 Reviewed [file giac019_reviewer_1_report_original_submission.pdf]

Reviewer Report

Title: A high-quality assembled genome and its comparative analysis decode the adaptive molecular mechanism of the number one Chinese cotton variety CRI-12

Version: Original Submission **Date:** 11/18/2021

Reviewer name: Sunil Kumar Sahu, PhD.,

Reviewer Comments to Author:

Cotton (Gossypium hirsutum L.) is the third largest oilseed crop in the world and also a source of relatively high-quality protein. In this study, the author reports the first and high-quality genome map of a widely farmed upland cotton CRI-12, which could help researchers better understand crop domestication, evolution, and diversification, as well as find new domestication-related genes that impart agronomically useful features in future breeding efforts. I found the manuscript to be interesting and well-written. The objective of the paper is clear and unambiguous. The article is well structured, the methodology is correct and suitable for the realization of the paper objectives. The dataset generated by the authors appears to be of good quality and I anticipate that it will be very useful for the research community. English language and style of the whole text are correct with rather minor drawbacks. However, before accepting the manuscript in its current form, I have several concerns and would suggest some minor revisions as follows:

1. Assembly: The authors used Pacbio + HiC to generate a high-quality genome assembly. Although they described the details regarding Pacbio data assembly. I couldn't find much details regarding how the HiC scaffolding was performed
2. The authors used different methods to evaluate the quality of the assembled genomes and obtained good results. Although the BUSCO score is good. It is better to use additional software to evaluate this genome quality. e.g., LTR Assembly Index (LAI), an important criterion for the genome quality nowadays, particularly for genome with a high percentage of TEs.
3. Fig. 1b and 1d. The font size is too small to read the content.
4. DNA extraction method lack reference citation.
5. Several methods are presented in too much details. If the original references are there, the only brief descriptions would be enough with relevant citations.
6. Figure S1a. The content of the certificate should be translated in English and described in legends for non-chinese readers.
7. I was wondering if there is any full name of CRI? If yes, please describe in Introduction section
8. Line 578. Check the spelling/grammar and rewrite the sentence "And the remain data…"
9. Check the image quality of the Supplementary figures.

Methods

Are the methods appropriate to the aims of the study, are they well described, and are necessary controls included? Choose an item.

Conclusions

Are the conclusions adequately supported by the data shown? Choose an item.

Reporting Standards

Does the manuscript adhere to the journal's guidelines on [minimum standards of reporting?]() Choose an item.

Choose an item.

Statistics

Are you able to assess all statistics in the manuscript, including the appropriateness of statistical tests used? Choose an item.

Quality of Written English

Please indicate the quality of language in the manuscript: Choose an item.

Declaration of Competing Interests

Please complete a declaration of competing interests, considering the following questions:

- Have you in the past five years received reimbursements, fees, funding, or salary from an organisation that may in any way gain or lose financially from the publication of this manuscript, either now or in the future?
- Do you hold any stocks or shares in an organisation that may in any way gain or lose financially from the publication of this manuscript, either now or in the future?
- Do you hold or are you currently applying for any patents relating to the content of the manuscript?
- Have you received reimbursements, fees, funding, or salary from an organization that holds or has applied for patents relating to the content of the manuscript?
- Do you have any other financial competing interests?
- Do you have any non-financial competing interests in relation to this paper?

If you can answer no to all of the above, write 'I declare that I have no competing interests' below. If your reply is yes to any, please give details below.

I declare that I have no competing interests

I agree to the open peer review policy of the journal. I understand that my name will be included on my report to the authors and, if the manuscript is accepted for publication, my named report including any attachments I upload will be posted on the website along with the authors' responses. I agree for my

report to be made available under an Open Access Creative Commons CC-BY license (http://creativecommons.org/licenses/by/4.0/). I understand that any comments which I do not wish to be included in my named report can be included as confidential comments to the editors, which will not be published.

Choose an item.

To further support our reviewers, we have joined with Publons, where you can gain additional credit to further highlight your hard work (see: https://publons.com/journal/530/gigascience). On publication of this paper, your review will be automatically added to Publons, you can then choose whether or not to claim your Publons credit. I understand this statement.

Yes Choose an item.
