# Supplementary material for: A high-quality assembled genome and its comparative analysis decode the adaptive molecular mechanism of the number one Chinese cotton variety CRI-12
Source: Gigascience. 2022 Apr 1;11:giac019. doi: 10.1093/gigascience/giac019 (PMC8975723; doi:10.1093/gigascience/giac019)
Supplement: giac019_Reviewer_2_Report_Original_Submission — Fatemeh Maghuly -- 11/20/2021 Reviewed [file giac019_reviewer_2_report_original_submission.pdf]

Reviewer Report

Title: A high-quality assembled genome and its comparative analysis decode the adaptive molecular mechanism of the number one Chinese cotton variety CRI-12

Version: Original Submission **Date:** 11/20/2021

Reviewer name: Fatemeh Maghuly

Reviewer Comments to Author:

Lu et al. have provided a high-quality reference genome of the Chinese No.1 cotton variety CRI-12 using the PacBio single-molecule long reads combined with Illumina short reads and Hi-C sequencing technologies.
The assembly is of high quality and was used for comparative genomics analysis. The approaches are technically sound, and adequate details are provided on the assembly and annotation of this genome. Furthermore, they used transcriptomic for genome annotation.
The amount of experimental work is high, and the overall quality of the analyses appears to be appropriate. However, I have a few minor concerns I feel should be addressed before this manuscript is published.

- The manuscript would significantly benefit if the Author removes repeated sentences throughout the manuscript (e.g. lines 113-114 and 123-124 or Lines 81-82 and 85-86).
- The authors described results related to methylation and SVs like CNVs or PVA, but their descriptions are not in Material and methods.
- Please add the number of biological replicates.
- The discussion is like a summary; therefore, I would suggest adding a conclusion instead.
- Lines 54-59: Please modify and add as a new paragraph.
- LineS 77-78: Please modify for clarification.
- Line 84: Please remove "finally" and modify the sentence.
- Lines 254-256: Please modify for clarification.
- Line 267: "… two high-quality…" could you please add the name
- Line 521: Could you please add the name of 14 species.
- Please clarify if "figure 3c" is from previous or current work.
- Line 548: Please add the full name for "PTF"?
- Please spell out all abbreviations and botanical names for the first time in the main text; after that, you can use the abbreviation and the short term.
- Latin name should be written in italics.

Methods

Are the methods appropriate to the aims of the study, are they well described, and are necessary controls included? Choose an item.

Conclusions

Are the conclusions adequately supported by the data shown? Choose an item.

Reporting Standards

Does the manuscript adhere to the journal's guidelines on minimum standards of reporting? Choose an item.

Choose an item.

Statistics

Are you able to assess all statistics in the manuscript, including the appropriateness of statistical tests used? Choose an item.

Quality of Written English

Please indicate the quality of language in the manuscript: Choose an item.

Declaration of Competing Interests

Please complete a declaration of competing interests, considering the following questions:

- Have you in the past five years received reimbursements, fees, funding, or salary from an organisation that may in any way gain or lose financially from the publication of this manuscript, either now or in the future?
- Do you hold any stocks or shares in an organisation that may in any way gain or lose financially from the publication of this manuscript, either now or in the future?
- Do you hold or are you currently applying for any patents relating to the content of the manuscript?
- Have you received reimbursements, fees, funding, or salary from an organization that holds or has applied for patents relating to the content of the manuscript?
- Do you have any other financial competing interests?
- Do you have any non-financial competing interests in relation to this paper?

If you can answer no to all of the above, write 'I declare that I have no competing interests' below. If your reply is yes to any, please give details below.

'I declare that I have no competing interests'

I agree to the open peer review policy of the journal. I understand that my name will be included on my report to the authors and, if the manuscript is accepted for publication, my named report including any attachments I upload will be posted on the website along with the authors' responses. I agree for my

report to be made available under an Open Access Creative Commons CC-BY license (http://creativecommons.org/licenses/by/4.0/). I understand that any comments which I do not wish to be included in my named report can be included as confidential comments to the editors, which will not be published.

Choose an item.

To further support our reviewers, we have joined with Publons, where you can gain additional credit to further highlight your hard work (see: https://publons.com/journal/530/gigascience). On publication of this paper, your review will be automatically added to Publons, you can then choose whether or not to claim your Publons credit. I understand this statement.

Yes Choose an item.
